# In silico integration of disease resistance QTL, genes and markers with the *Brassica juncea* physical map

**DOI:** 10.1007/s11032-022-01309-5

**Published:** 2022-06-27

**Authors:** Fabian Inturrisi, Philipp E. Bayer, Aldrin Y. Cantila, Soodeh Tirnaz, David Edwards, Jacqueline Batley

**Affiliations:** School of Biological Sciences and Institute of Agriculture, University of Western Australia, Perth, WA Australia

**Keywords:** Disease resistance genes, Nucleotide-binding site–leucine-rich repeat (NLR), Quantitative trait locus (QTL), Receptor-like protein (RLP), Receptor-like protein kinase (RLK), *Brassica juncea*

## Abstract

*Brassica juncea* (AABB), Indian mustard, is a source of disease resistance genes for a wide range of pathogens. The availability of reference genome sequences for *B. juncea* has made it possible to characterise the genomic structure and distribution of these disease resistance genes. Potentially functional disease resistance genes can be identified by co-localization with genetically mapped disease resistance quantitative trait loci (QTL). Here we identify and characterise disease resistance gene analogs (RGAs), including nucleotide-binding site–leucine-rich repeat (NLR), receptor-like kinase (RLK) and receptor-like protein (RLP) classes, and investigate their association with disease resistance QTL intervals. The molecular genetic marker sequences for four white rust (*Albugo candida*) disease resistance QTL, six blackleg (*Leptosphaeria maculans*) disease resistance QTL and *BjCHI1*, a gene cloned from *B. juncea* for hypocotyl rot disease, were extracted from previously published studies and used to compare with candidate RGAs. Our results highlight the complications for the identification of functional resistance genes, including the duplicated appearance of genetic markers for several resistance loci, including *Ac2(t)*, AcB1-A4.1, AcB1-A5.1, *Rlm6* and *PhR2* in both the A and B genomes, due to the presence of homoeologous regions. Furthermore, the white rust loci, *Ac2(t)* and AcB1-A4.1, mapped to the same position on chromosome A04 and may be different alleles of the same gene. Despite these challenges, a total of nine candidate genomic regions hosting 14 RLPs, 28 NLRs and 115 RLKs were identified. This study facilitates the mapping and cloning of functional resistance genes for applications in crop improvement programs.

**Supplementary Information:**

The online version contains supplementary material available at 10.1007/s11032-022-01309-5.

## Introduction

*Brassica juncea*, commonly known as Indian mustard, is extensively cultivated, with a total of 952,727 hectares producing 728,931 tonnes across 25 countries in 2018 (FAO 2020). However, its production is limited by several diseases, including blackleg (*Leptosphaeria maculans, L. biglobosa*), Sclerotinia stem rot (*Sclerotinia sclerotiorum*), white rust (*Albugo candida)*, Alternaria blight (*Alternaria brassicae, A. brassicicola, A. raphani)*, downy mildew (*Hyaloperonospora brassicae*), white leaf spot (*Pseudocercosporella capsellae*) and powdery mildew (*Erysiphe cruciferarum*) (Oram et al. 1999; Pradhan and Pental 2011; Edwards et al. 2007; Inturrisi et al. 2021). Traditional disease management strategies include fungicide application and seed treatments, crop rotation and the use of resistant cultivars. However, the effectiveness of resistant cultivars can break down over time due to changing pathogen populations and the diversity of avirulence genes (Zhang et al. 2015; Rouxel and Balesdent 2017). For example, the breakdown of blackleg resistance in commercial cultivars of *B. napus* has been reported in Australia and Canada (Van De Wouw et al. 2016; Rouxel and Balesdent 2017; Van de Wouw et al. 2014; Zhang et al. 2015). Because of the potential to break down resistance, it is important to expand the sources of resistance for introgression into commercial *B. napus* cultivars. Sources of germplasm for resistance breeding have been identified in several *B. juncea* genotypes coming from the major producing countries, such as Australia, Canada, China and India, for resistance to *L. maculans*, *S. sclerotium* and *A. candida* (Li et al. 2006, 2007a, b, 2008a, b), and *B. juncea* has proven to be a valuable source of resistance genes for introgression into other *Brassica* crops, such as canola (*B. napus*) (Inturrisi et al. 2021).

Plant resistance gene analogs (RGAs) play an important role in plant resistance response against pathogens (Zhang et al. 2020; Sekhwal et al. 2015). The nucleotide-binding site–leucine-rich repeats (NLR), receptor-like kinases (RLK) and receptor-like proteins (RLP) are the main classes of RGAs (Zipfel 2014, 2008; Kim et al. 2012; Stotz et al. 2014). In a typical NLR gene, the NBS and LRR domains are located in the middle and the C-terminus of the gene respectively (Meyers et al. 1999; Xiao et al. 2001; Shao et al. 2014). The remaining structure of NLR proteins consists of three main domains at the N-terminus; the TIR-NBS-LRR (TNL) class is characterized by a toll/interleukin-1 receptor domain; the CC-NBS-LRR (CNL) class contains the coiled-coil domain; and the RPW8-NBS-LRR (RNL) class contains the resistance to powdery mildew 8 (RPW8) domain. Different types of RLKs include leucine-rich repeat–receptor-like kinases (LRR-RLKs), the largest gene family of RLKs and are highly conserved, and the less conserved lysin motif–receptor-like kinases (LysM-RLKs) (Gust et al. 2012; Wan et al. 2008; Zeng et al. 2012). In contrast, RLPs have an extracellular domain, a transmembrane domain and a short cytosolic domain without a signalling domain. Some of the main types of RLPs include leucine-rich repeat–receptor-like proteins (LRR-RLPs) (Jones et al. 1994; Jehle et al. 2013) and lysin motif–receptor-like proteins (LysM-RLPs) (Willmann et al. 2011). These genes have been identified and studied across *Brassica* species and *Brassica* pangenomes (Inturrisi et al. 2020; Yang et al. 2021; Bayer et al. 2019; Dolatabadian et al. 2020), as well as wild and cultivated species of the Brassicaceae family (Tirnaz et al. 2020).

Genetic mapping in plants has been used extensively to identify genetic regions associated with traits (Rafalski 2002; Tanksley et al. 1989; Mohan et al. 1997; Xu et al. 2017). The availability of plant reference genomes and pangenomes allows for translation of these genetic loci to genomic regions and the identification of candidate gene variations underlying heritable traits (Dolatabadian et al. 2020; Hurgobin and Edwards 2017; Bayer et al. 2019), and the availability of the *B. juncea* genome (Yang et al. 2016) allows the identification of candidate disease resistance genes underlying previously identified disease resistance quantitative trait loci (QTL) in this species. Here we performed in silico analysis to identify disease resistance QTL from published literature and map genetic locations to *B. juncea*, based on flanking molecular genetic markers. Candidate genes for disease resistance were identified in the QTL intervals, including several NLR, RLP and RLK genes. These genes provide candidates for further assessment and validation for their role in defence against these important diseases.

## Materials and methods

### Genomic resources

Molecular genetic markers associated with resistance to infection by *L. maculans* and *A. candida* pathogens, and *BjCHI1* resistance for hypocotyl rot disease in *B. juncea* were identified in published literature (Tables S1, S2, S3). The sequence of the markers and genes were downloaded from the literature, the NCBI (https://www.ncbi.nlm.nih.gov/) or TAIR (https://www.arabidopsis.org/index.jsp) websites (Tables S1, S2, S3).

The list of RGAs, including NLRs, RLKs and RLPs, was extracted from previous studies (Tirnaz et al. 2020; Yang et al. 2021; Inturrisi et al. 2020). All classes of RGAs were identified based on their domain structure using the RGAugury pipeline (Li et al. 2016b). RLKs were further classified into three types; ‘RLK-LRR’, ‘RLK-LysM’ and ‘RLK-other-receptor’. RLPs were classified into two types; ‘RLP-LRR’ and ‘RLP-LysM’ and NLRs were classified to ‘NL’, ‘TN’, ‘TNL’, ‘CNL’, ‘N’ and ‘other’ subclasses. The graphical representation of NLR, RLK and RLP genes was visualised using Mapchart V2.3 (Voorrips 2002).

### Characterisation of published resistance QTL to the physical position on the reference genome

The physical chromosomal positions of the disease-associated molecular genetic markers were determined by comparing the sequences (Tables S1–S3) with the reference *B. juncea* genome v1.5 (Yang et al. 2016) on the Brassica database (BRAD) website (http://brassicadb.org/brad/blastPage.php). If a marker could not be placed on a pseudomolecule or a contig, it was removed from further analysis.

Previously predicted NLR, RLK and RLP genes (Tirnaz et al. 2020; Yang et al. 2021; Inturrisi et al. 2020) were assigned as candidate resistance genes if positioned within the flanking markers of a QTL interval or, alternatively, positioned 1 Mbp region upstream and downstream of the marker.

## Results

### Integration of QTL for disease resistance in *Brassica juncea*

#### White rust

In silico mapping for disease resistance has been conducted for white rust disease in *B. juncea*, where four QTL (Table S4) were identified. Marker sequences were available for white rust resistance loci: *Ac2(t)*, *Acr*, AcB1-A5.1 and AcB1-A4.1 (Table S1). Four white rust resistance QTL had marker sequences available to locate the genomic region of the QTL. Sequences for the primer pair of ILP marker At5g41560 (Panjabi et al. 2008), and RAPD primers OPB06 (OPB06_1000_) and OPN01 (OPN01_1000_) were obtained from (Rajaseger et al. 1997; Ananga et al. 2006; Solmaz et al. 2010). In addition, *BjCHI1*, a gene cloned from *B. juncea* for hypocotyl rot resistance, was identified (Table S3).

Genomic sequences for *A. thaliana* genes At2g34510, At2g36360, At5g41560 and At5g41940 from which markers for white rust resistance loci AcB1-A4.1 and AcB1-A5.1 (Panjabi-Massand et al. 2010) were derived from the TAIR website. The DNA sequence of all four genes was compared with the *B. juncea* reference to identify their approximate position.

The same white rust QTL represent the genes *Acr* and *Ac2*_*1*_. These genes were identified using the same mapping population from the crossing of susceptible J90-4317 and resistant J90-2733 (Prabhu et al. 1998; Cheung et al. 1998; Somers et al. 1999). In addition, it was suggested that *Ac2*_*1*_ and *Ac2(t)* are two separate loci despite the absence of polymorphism for markers between the two cultivars identified with *Ac2*_*1*_ and *Ac2(t)* (Mukherjee et al. 2001).

There were several markers and QTL that could not be analysed further due to unavailable sequence information, including RFLP markers for *Acr*; X140a, X42 and X83 (Cheung et al. 1998).

#### Blackleg

Ten blackleg QTL were identified in *B. juncea* (Table S5), while the flanking markers were unavailable for several QTL. Marker sequences were available for blackleg resistance loci, Rlm6, LMJR1, LMJR2, rjlm2 and PhR2, as well as a locus without an identifying name (Table S2). Sequences for the primer pair of ILP marker OPG02 (OPG02.800), OPT01 (OPT01.800), OPI01 (OPI01-HaeIII) and OPU9 were sourced from (Kumar et al. 2010; Srivastava et al. 2014; Delourme et al. 1994; Struss et al. 1996). The blackleg QTL, *LMJR1* and *LMJR2*, were flanked by one RFLP and one SSR; however, the SSR sequences (*LMJR1*, sB31143F; *LMJR2*, sB1534) were unavailable. The flanking RFLP markers pN199RV and pN120cRI were found on the NCBI website as pN199 (GenBank: CZ692853.1) and pN120 (GenBank: CZ692836.1), respectively, where the RFLP were named differently depending on the linkage map, species and publication. In addition, the SSR markers positioned in the same linkage group to SSRs flanking the *LMJR1* and *LMJR2* resistance loci were analysed due to the unavailability of sBb31143F and sB1534. Additional literature (Fredua-Agyeman et al. 2014; Nelson et al. 2009; Chen et al. 2013; Navabi et al. 2010, 2011) was investigated for the *Brassica* B genome linkage groups that were used in Christianson et al. (2006) to determine the SSRs with available marker sequence found in the same *Brassica* B genome linkage group.

In some instances, multiple blackleg disease QTL were found to represent the same gene, for example *Rlm6* and *Jlm1* (Chèvre et al. 1997; Balesdent et al. 2002; Brun et al. 2000; Fudal et al. 2007) due to a change in nomenclature. Four RFLP and three AFLP markers were linked to the blackleg resistance gene, *PhR2*, where an RFLP (RP1513) and AFLP (S7G4) marker were converted to PCR-based STS markers linked to the same resistance gene and mapped to the same position in linkage maps (Plieske and Struss 2001).

Sequence information of three RFLP markers for *PhR2* (pRP1457.H, pRP1513.E, pRP1602.H) (Plieske et al. 1998), RFLP markers linked to three resistance loci (Dixelius and Wahlberg 1999) and one unnamed RFLP associated to resistance locus, *LmBR1* (Dixelius 1999), were not available and they were excluded from the analysis.

### Physical mapping of candidate disease resistance genes in *Brassica juncea*

#### White rust

**Fig. 1:**
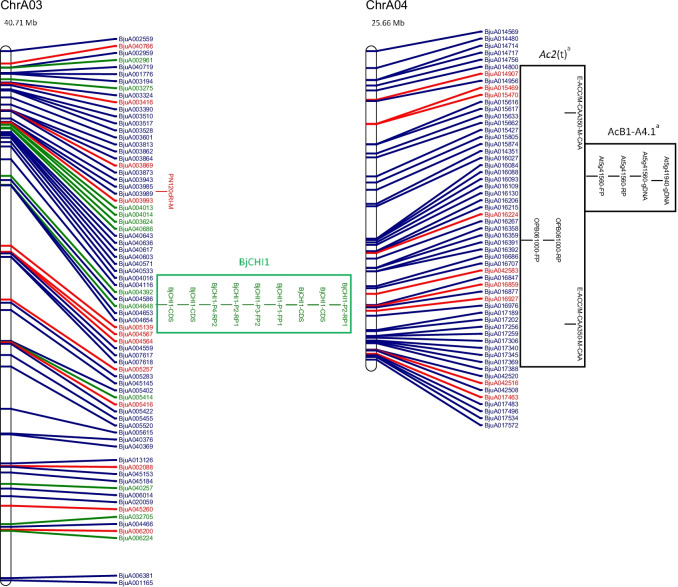

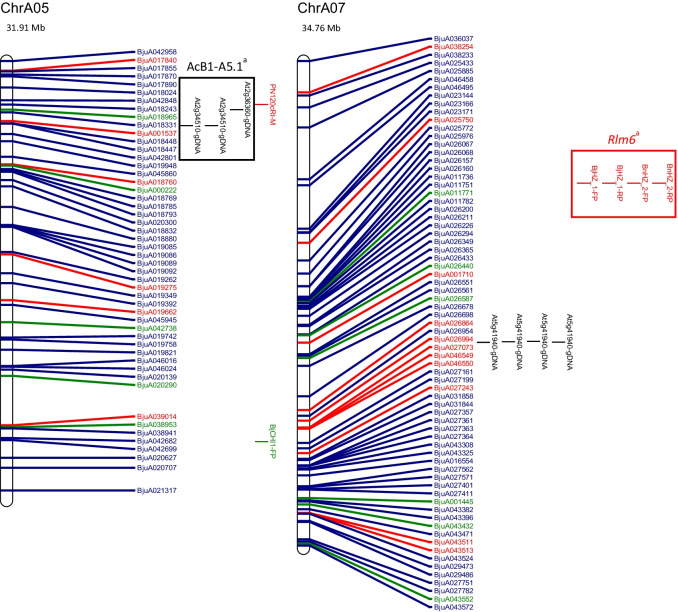

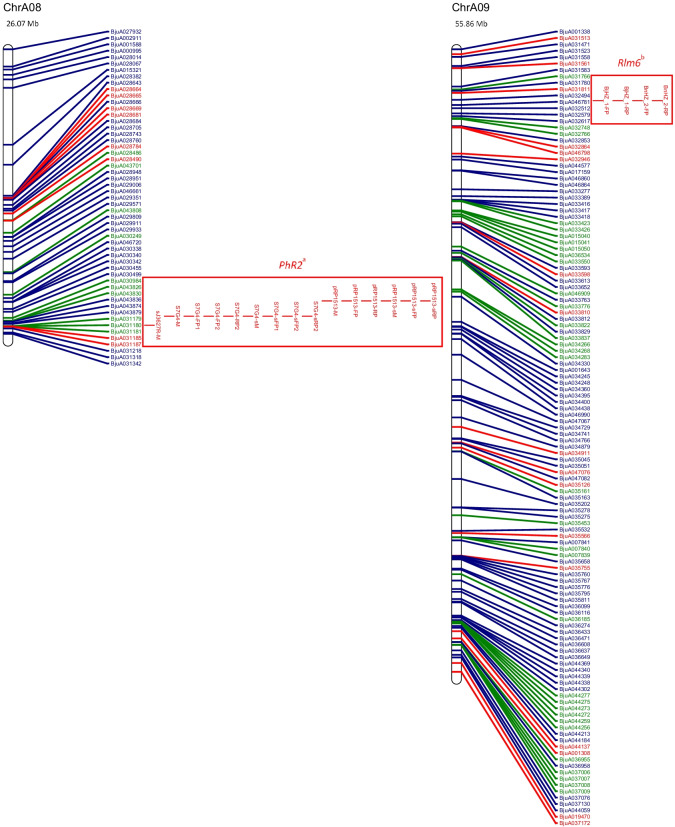

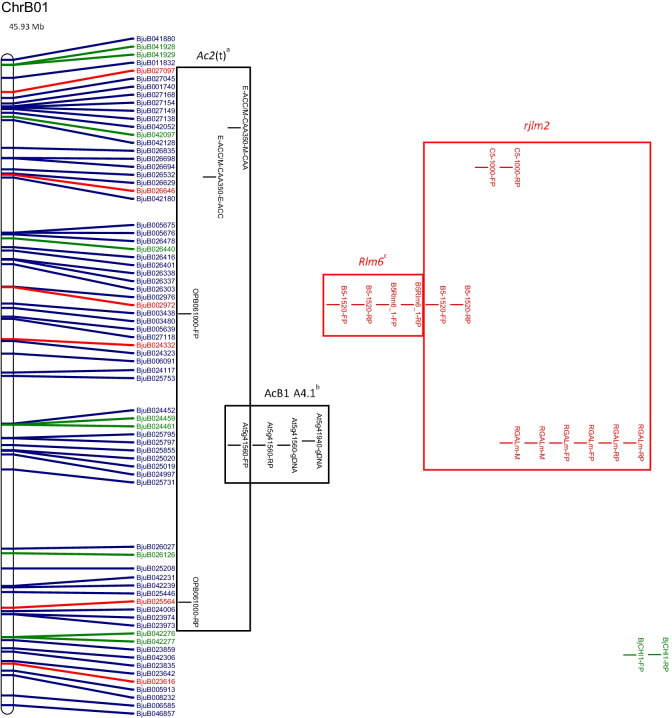

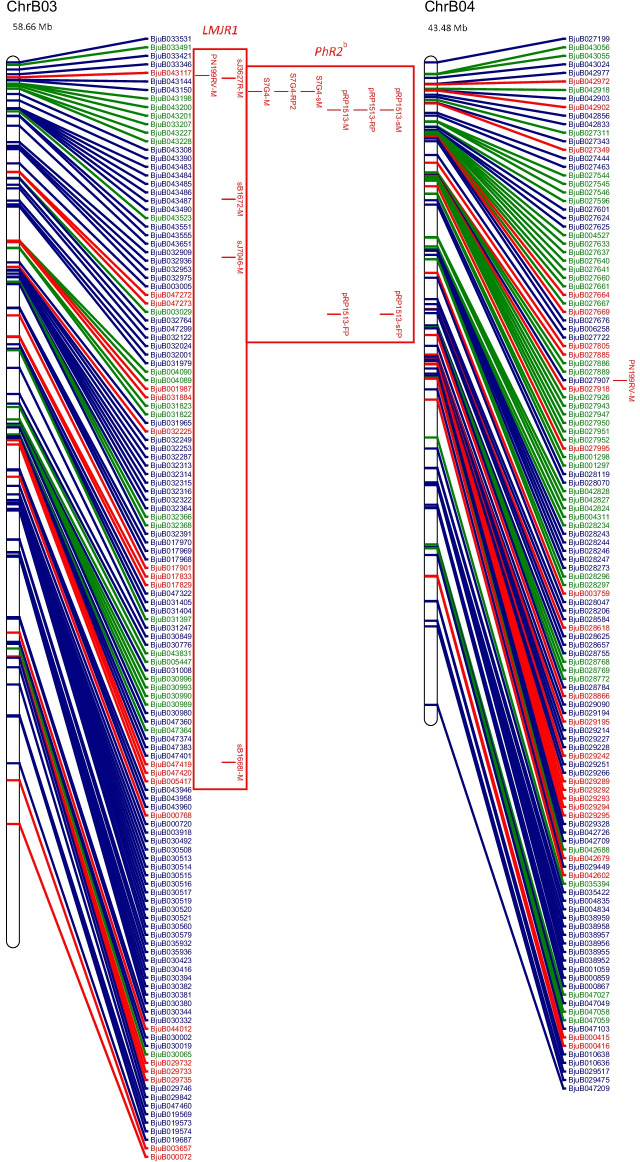

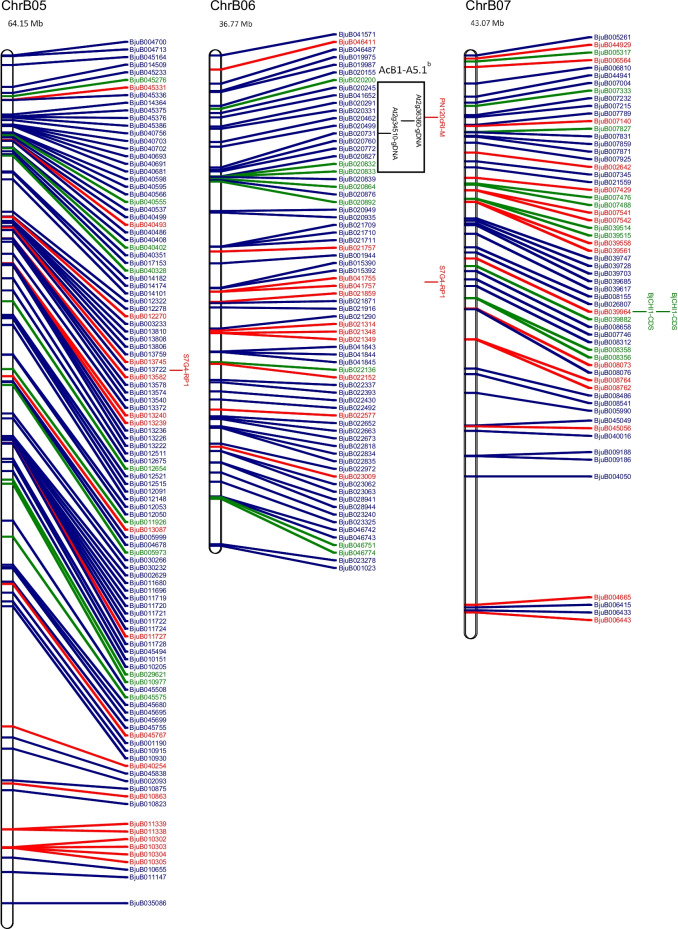

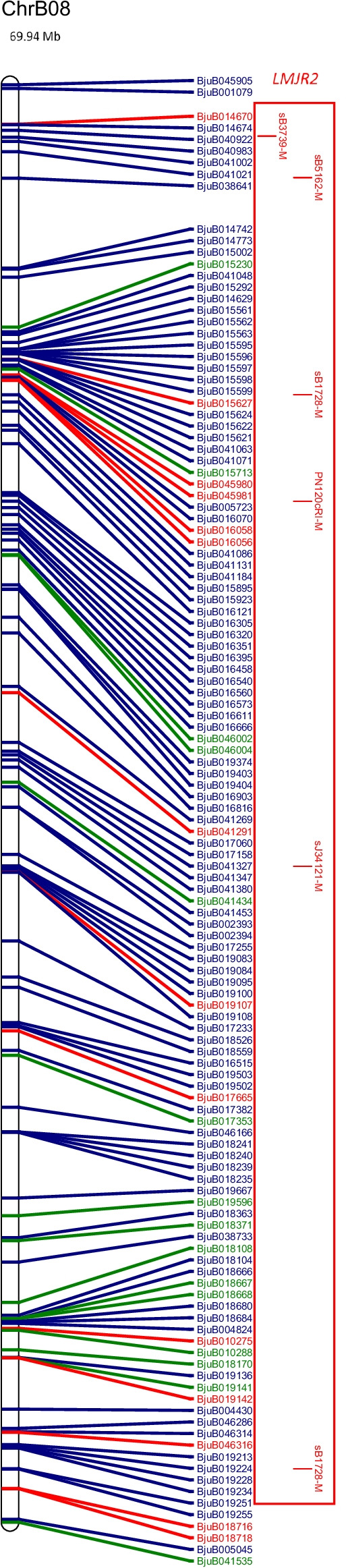
Physical distribution of markers linked to disease resistance in *B. juncea* along with resistance genes on the physical map of *B. juncea*. *B. juncea* chromosomes (Chr) are represented as white bars with resistance gene names shown on the right-hand side. The physical positions of genetic markers are indicated as coloured horizontal bars and named for white rust (black), blackleg (red) and hypocotyl (green). The suffixes ‘-M’, ‘-FP’ and ‘-RP’ indicate that the physical position was based on the sequence of the marker, a forward primer or a reverse primer, respectively. Regions of sequence identity for markers and genes are placed into a box with loci names. Letter subscript for loci names indicates more than one region of interest for a particular resistance locus or gene. Different colours and font of gene names reflect different classes of RGA; NLRs (dark green), RLKs (dark blue), RLPs (red). The chromosome size is shown above the chromosome bar (Mb)

The physical map and distribution of previously predicted RGAs (Yang et al. 2021; Inturrisi et al. 2020; Tirnaz et al. 2020) on *B. juncea* chromosomes were produced (Figure S1, Fig. 1). An uneven distribution of NLRs, RLPs and RLKs was observed between *B. juncea* chromosomes (Figure S1). For example, RLP-LysM genes were only found on chromosomes A06 and B03 and the majority of RLKs were located on chromosomes A03, B02, B03, B05, B08 and ‘unknown’.

White rust resistance QTL for AcB1-A4.1, AcB1-A5.1 and *Ac2(t)* were located on the A genome chromosomes A04, A05 and A04, respectively (Fig. 1). Flanking markers for QTL AcB1-A4.1(B01), AcB1-A5.1(B06) and *Ac2(t)*(B01) aligned with regions on the B genome chromosomes. There were no significant matches for the decamer primers for RAPD markers WR2 and WR3, which are flanking markers for *Acr* (= *Ac2*_*1*_); hence, no genomic position could be determined.

The sequence of several flanking markers for white rust QTL had multiple matches on different chromosomes (Fig. 1). The sequences of E-ACC and M-CAA, for the marker E-ACC/M-CAA_350_ for a white rust resistance locus *Ac2(t)*, were found on chromosome B01. However, only M-CAA for marker E-ACC/M-CAA_350_ was identified on chromosome A04 and the distance between the primers was 3,466,012 bp on chromosome B01, which is more than the estimated size of E-ACC/M-CAA_350_, 350 bp.

Two genomic locations were found on chromosome A05 for the white rust resistance QTL, AcB1-A5.1. These had QTL interval sizes of 1,098,849 bp and 1,102,681 bp and were positioned 3832 bp apart. The flanking markers for AcB1-A4.1 were placed on chromosomes A04 and B01. The flanking marker At5g41940 for AcB1-A4.1 had top BLAST hits on chromosome A07 and Contig1207, although the other flanking marker At5g41560 failed to have top BLAST hits in these locations. It was also found with other markers for QTL regions that top BLAST hits were found on multiple chromosomes and multiple positions of the same chromosome (Fig. 1).

Candidate resistance genes were mapped within the locus and 1 Mb downstream and upstream from the white rust resistance (Table 1). RLKs were dominant in all QTL. The white rust QTL linked to AcB1-A4.1, B01, has the smallest size (304,083 bp) and lowest number of RGA (3) among all QTL. In total, there were five genomic regions of interest with 2 RLPs, 3 NLRs and 27 RLKs (Table 1).

**Table 1:** Candidate resistance genes mapped in loci for disease resistance from published literature

Chromosome	Locus or gene	Locus interval position (loci interval ± 1 Mb) (bp)	Locus interval size (bp)	Number of NBS-LRR(s)^1^	Number of RLK(s)^1^	Number of RLP(s)^1^	Candidate NBS-LRR(s)^1^	Candidate RLK (s)^1^	Candidate RLP(s)^1^
ChrA03	BjCHI1 (CDS)	20,353,574–20,355,565 (19,353,574–21,355,565)	1991	0	0	0	N.A	N.A	N.A
ChrA04	AcB1-A4.1^a^	10,446,467–10,808,704 (9,446,467–11,808,704)	362,237	0	3	0	N.A	BjuA015427, BjuA015805, BjuA015874	N.A
ChrA05	AcB1-A5.1^a^	4,795,221–5,894,070 (3,795,221–6,894,070)	1,098,849	1	6	1	BjuA018965	BjuA042848, BjuA018243, BjuA018331, BjuA018448, BjuA018447, BjuA042801	BjuA001537
	AcB1-A5.1^a*^	4,795,221–5,897,902 (3,795,221–6,897,902)	1,102,681	1	6	1	BjuA018965	BjuA042848, BjuA018243, BjuA018331, BjuA018448, BjuA018447, BjuA042801	BjuA001537
ChrA08	*PhR2*^a^	22,485,767–23,843,799 (21,485,767–24,843,799)	1,358,032	7	9	2	BjuA030249, BjuA030984, BjuA043826, BjuA043830, BjuA031179, BjuA031180, BjuA031181	BjuA046720, BjuA030338, BjuA030340, BjuA030342, BjuA030455, BjuA030499, BjuA043836, BjuA043874, BjuA043879	BjuA031185, BjuA031187
ChrB01	AcB1-A4.1^b^	27,117,536–27,421,619 (26,117,536–28,421,619)	304,083	0	6	0	N.A	BjuB025795, BjuB025797, BjuB025855, BjuB025020, BjuB025019, BjuB024997	N.A
ChrB03	*LMJR1*	1,498,805–9,675,185 (498,805–10,675,185)	8,176,380	9	27	3	BjuB033491, BjuB043198, BjuB043200, BjuB043201, BjuB033207, BjuB043227, BjuB043228, BjuB043523, BjuB003029	BjuB033531, BjuB033421, BjuB033346, BjuB043144, BjuB043150, BjuB043308, BjuB043390, BjuB043483, BjuB043484, BjuB043485, BjuB043486, BjuB043487, BjuB043490, BjuB043551, BjuB043555, BjuB043651, BjuB032909, BjuB032936, BjuB032953, BjuB032975, BjuB003005, BjuB032764, BjuB047299, BjuB032122, BjuB032024, BjuB032001, BjuB031979	BjuB043117, BjuB047272, BjuB047273
	*PhR2*^b^	2,554,162–3,778,538 (1,554,162–4,778,538)	1,224,376	7	12	1	BjuB043198, BjuB043200, BjuB043201, BjuB033207, BjuB043227, BjuB043228, BjuB043523	BjuB043144, BjuB043150, BjuB043308, BjuB043390, BjuB043483, BjuB043484, BjuB043485, BjuB043486, BjuB043487, BjuB043490, BjuB043551, BjuB043555	BjuB043117
ChrB06	AcB1-A5.1^b^	5,226,533–6,156,115 (4,226,533–7,156,115)	929,582	1	6	0	BjuB020200	BjuB020245, BjuB041652, BjuB020291, BjuB020331, BjuB020462, BjuB020499	N.A
ChrB08	*LMJR2*	0–20,282,056 (0–21,282,056)	20,282,056	2	40	6	BjuB015230, BjuB015713	BjuB045905, BjuB001079, BjuB014674, BjuB040922, BjuB040983, BjuB041002, BjuB041021, BjuB038641, BjuB014742, BjuB014773, BjuB015002, BjuB041048, BjuB015292, BjuB014629, BjuB015561, BjuB015562, BjuB015563, BjuB015595, BjuB015596, BjuB015597, BjuB015598, BjuB015599, BjuB015624, BjuB015622, BjuB015621, BjuB041063, BjuB041071, BjuB005723, BjuB016070, BjuB041086, BjuB041131, BjuB041184, BjuB015895, BjuB015923, BjuB016121, BjuB016305, BjuB016320, BjuB016351, BjuB016395, BjuB016458	BjuB014670, BjuB015627, BjuB045980, BjuB045981, BjuB016058, BjuB016056

*N.A.* not available.

^1^Information provided for loci interval ± 1 Mb.

#### Blackleg

Markers for blackleg resistance QTL were identified on several chromosomes (Fig. 1). Markers for *PhR2* were positioned on chromosomes A03 and B03 with interval sizes of 1,358,032 bp and 1,224,376 bp, respectively. Markers for *LMJR1* were positioned on chromosome B03 with loci interval size of 8,176,380 bp based on the set of SSRs utilised in the study (Christianson et al. 2006). *PhR2* overlaps with *LMJR1* on chromosome B03 at 2,554,162 bp–3,778,538 bp. In addition, the genomic region of interest for *LMJR2* was identified using a similar approach to *LMJR1* where SSR markers of the same linkage group of the SSR that was linked to *LMJR2* were positioned on chromosome B08, with a locus interval of 20,282,056 bp. The markers for *Rlm6* were located on several chromosomes: A07, A09 and B01 (Fig. 1). The RAPD and RFLP markers were found to be linked to *Rlm6* from a couple of studies (Chèvre et al. 1997; Barret et al. 1998). CAPS and SCAR markers that were linked to *Rlm6* were used for screening *B. napus* and *B. juncea* interspecific hybrid populations (Rashid et al. 2018). The CAPS markers, BnHz_2 and BjHz_1, did not provide a QTL region, unlike *r*_*j*_*lm2*, *PhR2*, *LMJR1* and *LMJR2* (Christianson et al. 2006; Plieske and Struss 2001; Saal and Struss 2005; Saal et al. 2004), and were found on chromosome A07 and A09. However, the SCAR markers for *Rlm6*, B5-1520 and B5Rlm6_1, were found on chromosome B01 and they were shown to be located within the *r*_*j*_*lm2* locus (Fig. 1). All three SCAR markers linked to *r*_*j*_*lm2*, B51520, C5-1000 and RGALm, were found on chromosome B01.

Similar to white rust, candidate resistance genes were mapped within the locus and 1 Mb downstream and upstream from the loci for blackleg (Table 1), and all QTL for resistance had the highest proportion of RLKs. Most QTL contained more NLRs than RLPs, expect for *LMJR2* on chromosome B08 (2 NLRs and 6 RLPs). Among all QTL, blackleg QTL linked to *LMJR2*, B08, has the largest size (20,282,056 bp) and highest number of RGA (48). Genomic regions of interest with a larger size interval tended to have a greater number of total RGAs. The SCAR markers, B5-1520 and B5Rlm6_1, were linked to *Rlm6*; however, there was no linkage map information provided by Rashid et al. (2018) and B5-1520 was the same SCAR marker utilised for *r*_*j*_*lm2*. *Rlm6* was located on chromosome B01 using the SCAR markers, B5-1520 and B5Rlm6_1, with an interval of 1467 bp where BjuB003452 overlapped with the interval of the markers (Rashid et al. 2018). Analysis of the locus interval along with 1 MB upstream and downstream mapped four RLK genes (BjuB003438, LRR-RLK; BjuB003480, LysM-RLK; BjuB005639, Other-receptor-RLK BjuB027118, LRR-RLK). There were two RLPs outside of this region for analysis (BjuB002972, LRR-RLP, B01, 16,299,149–16,300,492; BjuB024332, LRR-RLP, B01, 19,952,790–19,957,271). For blackleg resistance, in total, there were four genomic regions of interest with 12 RLPs, 25 NLRs and 88 RLKs (Table 1).

#### Hypocotyl rot

*BjCHI1* (GenBank accession no. AAF02299), a chitinase gene for hypocotyl rot resistance, was mapped in an unnamed *B. juncea* genotype using primers derived from an *Arabidopsis* chitinase gene (Zhao and Chye 1999). The coding sequence for *BjCHI1* was available and was BLASTed against the *B. juncea* reference genome and found to be positioned on chromosome A03, 20,353,574–20,355,565 bp, with a coding sequence length of 1991 bp on the reference genome. An annotated gene from the *B. juncea* reference genome, BjuA012108, was shown to overlap with the physical position of the coding sequence for *BjCHI1* on chromosome A03. The physical position of BjuA012108 was on chromosome A03 at 20,353,662 to 20,355,562 bp with a gene length of 1901 bp and consisting of two exons. BjuA012108 was not previously identified as a RGA in *B. juncea*; however, it is reported as a member of the glycosyl hydrolases gene family which is also involved in plant defence mechanisms against microbes and herbivores (Mir et al. 2020).

## Discussion

In this study, the association of RLKs, RLPs and NLRs, the main classes of RGAs, with *B. juncea* resistance QTL of white rust, blackleg and hypocotyl rot diseases were investigated. Similar genetic mapping studies were previously performed across various crop species for the identification of functional resistance genes. For instance, Sagi et al. (2017) validated candidate NLR genes for ascochyta blight resistance in chickpea that were co-localised within the QTL interval from previously published studies using qRT-PCR among three cultivars at four different time points. These candidate NLRs were selected for validation after the identification of NLR genes from the chickpea reference genome and physical positioning of the flanking markers for known disease resistance QTL in chickpea. Candidate genes have been identified in *Brassica* species through genetic analysis for disease resistance against Sclerotinia stem rot in *B. napus* (Wei et al. 2016; Wu et al. 2016), clubroot disease in *B. napus* (Li et al. 2016a) and *B. rapa* (Yu et al. 2017), blackleg disease in *B. napus* (Cantila et al. 2020; Tollenaere et al. 2012; Raman et al. 2016), yellow wilt disease in *B. oleracea* (Lv et al. 2014; Shimizu et al. 2015), turnip mosaic virus disease in *B. rapa* (Lv et al. 2015) and downy mildew in *B. rapa* (Yu et al. 2016). Wu et al. (2016) performed a comparative analysis for Sclerotinia stem rot resistance in *B. napus* where QTL identified in the study and previous studies aligned to the *B. napus* genome based on the physical position of the markers (Wu et al. 2016). There were 41 genes identified for Sclerotinia stem rot resistance among 12 *B. napus* chromosomes along with three chromosomal regions with multiple QTL (Wu et al. 2016). An integration analysis of QTL for Sclerotinia stem rot in *B. napus* was conducted by Li et al. (2015) where QTL from previous studies were aligned to the *B. napus* genome to identify 26 candidate NLRs. In addition, 4 and 7 NLRs were identified on conserved QTL regions located on *B. napus* chromosome A9 and C6, respectively (Li et al. 2015). An integration analysis for disease resistance in *B. juncea* was similar to approaches implemented by Wu et al. (2016) to identify candidate resistance genes. Here we mapped over 100 RGAs in *B. juncea* genome; however, not all of them are necessarily involved in a resistance response. In particular RLK and RLP genes are also involved in various, other biological processes (Sekhwal et al. 2015).

Here, QTL analysis indicates that the positions of the QTL markers did not always have hits on the same chromosomes. For example, for *Ac2(t)*, the forward primer for OPB06_1000_ was aligned to chromosomes A04 (e-value = 0.000297) and B01 (e-value = 0.001) although the reverse primer and the other flanking marker, E-ACC/M-CAA_350_, were not strongly aligned. This observation could be due to differences in the marker sequences between the lines they were identified in and the reference genome, or it could be problems with genome assemblies. For example, the wrong placement of a contig in the genome assembly or duplication may lead to the forward and reverse primers for a marker, or different markers underlying the QTL being found on different chromosomes. In addition, at the time that markers were developed for these QTL, the reference genome of *B. juncea* was not available and it was impossible to design primers for exact genomic physical positions.

Some QTL had positions on multiple chromosomes. For example, AcB1-A4.1 had a similar QTL region size of 362,237 bp and 304,083 bp on chromosomes A04 and B01, respectively. There were two QTL regions for AcB1-A5.1 on chromosome A05. In addition, At2g34510 and At2g36360 for AcB1-A5.1 were located on chromosome B06 at an interval size of 929,582 bp. The candidate resistance genes for AcB1-A4.1, AcB1-A5.1 and *Ac2(t)* were placed on the A genome chromosomes, A04, A05 and A04, respectively. Mapping QTL on more than one chromosome and often different sub-genomes may be caused by homoeologous regions between the A and B genome and result in the identification of orthologous and homogeneous resistance genes. This will highlight the importance of performing a genome-wide analysis and not only focus on the reported regions for a specific gene and marker to ensure all candidate genes have been considered.

In some cases, two different genes were mapped in the same position. For example, the disease locus of AcB1-A4.1 overlapped with *Ac2(t)* on chromosome A04, which could suggest two disease resistance loci on the same chromosome, or that AcB1-A4.1 and *Ac2(t)* were actually the same disease resistance locus. AcB1-A4.1 and *Ac2(t)* being the same disease resistance locus was suggested by Singh et al. (2015). This is a common challenge when different markers or populations are used and it is unclear whether loci are distinct genes, different alleles of the same gene or in fact the same gene.

Although it was found the majority of RGAs underlying the QTL were NLR genes and they play a major role in plant disease resistance (Meyers et al. 1999; McHale et al. 2006), most of the previously cloned resistance genes for white rust and blackleg resistance were reported as RLKs and RLPs. For instance, the resistance genes for blackleg resistance cloned from *Brassica* species, i.e. *Rlm2* and *LepR3*, were shown to encode extracellular leucine-rich receptor (eLRR) receptor-like proteins (RLPs) on chromosome A10 (Larkan et al. 2013, 2015). *LepR3* was annotated as Bra008930 in *B. rapa*, which was 1890 bp gene length, 851 amino acid length and motif structure predicted by InterProScan analysis to include a single peptide at the N-terminal, eLRR region, transmembrane motif and cytoplasmic C-terminal region at the C-terminal (Larkan et al. 2013). *Rlm2* was cloned in *B. napus* and had a protein motif structure to include a single peptide at the N-terminal, eLRR region, transmembrane motif and cytoplasmic C-terminal region at the C-terminal (Larkan et al. 2015). *Rlm2*, an LRR-RLP, was shown to interact with *At*SOBIR1, a LRR-RLK gene (Larkan et al. 2015), and *LepR3* was shown to interact with *BnSOBIR1*, a *B. napus* RLK (Ma and Borhan 2015). These highlight the importance of including RLKs and RLPs, in addition to NLRs for the identification of functional resistance genes.

Many of the QTL intervals were shown to contain clustered RGAs. This has been observed in previous studies, which found QTL located in a resistance gene–rich region containing NLR gene clusters that confer resistance to a number of different pathogens (Wang et al. 2010; Jeong et al. 2001). A clustered region of NLR genes has also been found to underly different QTL conferring fungal resistance in soybeans (Kang et al. 2012). This may have implications for disease resistance and be beneficial for resistance gene evolution.

The markers for resistance against the same race of pathogen isolates are possibly useful for further analysis between plant species, especially closely related plant species like diploid *B. nigra* and allotetraploid *B. juncea* which both possess the *Brassica* B genome. *B. juncea* has been shown to have resistance to multiple races (1, 3, 4, 7, 8, 9) of *Xanthomonas campestris*, the causal agent for black rot, where it was postulated that *B. juncea* have black rot resistance genes, *R1*, *R5* and possibly *R4* (Vicente et al. 2001; Jensen et al. 2010; Vicente and Holub 2013). The resistance genes for *R1* and *R4* were established to be single dominant genes, which correspond to avirulence genes *A1* and *A4*, respectively. It was also postulated that *B. juncea* cultivar Guangtou possessed resistance gene *Rc1*, which corresponds to avirulence gene *avrXccC* (*xopAH*) (He et al. 2007), and resistance genes *Rc1* and *Rc3* may be resistance genes *R1* and *R4*.

To conclude, with advances in whole-genome sequencing technologies and availability of crops genome sequence, it is important to perform genome-wide mapping studies of genes and QTL linked to agronomically important traits, such as disease resistance, to facilitate the identification and application of these genes in the breeding programme. Here we mapped nine genomic regions related to disease resistance in *B. juncea* where the regions carry14 RLPs, 28 NLRs and 115 RLKs. We also indicated and discussed a number of challenges that can affect the accuracy of gene identification, including identifying QTL markers in various locations across the genome, which can be observed as a result of the homologs genomic regions. Altogether, we expected the outcome assists and facilitates the identification of functional genes towards breeding improvements.

## Supplementary Information

Below is the link to the electronic supplementary material.

Supplementary file1 (DOCX 26 KB)

Supplementary file2 (DOCX 26 KB)

Supplementary file3 (DOCX 21 KB)

Supplementary file4 (DOCX 17 KB)

Supplementary file5 (DOCX 18 KB)

Supplementary file6 (PDF 289 KB)

## Data Availability

Access to the data were presented in Table S1, S2, S3, S4 and S5.
