# Supplementary material for: In silico integration of disease resistance QTL, genes and markers with the Brassica juncea physical map
Source: Mol Breed. 2022 Jun 27;42(7):37. doi: 10.1007/s11032-022-01309-5 (PMC10248627; doi:10.1007/s11032-022-01309-5)
Supplement: Supplementary file 1 — Supplementary file1 (DOCX 26 KB) [file 11032_2022_1309_MOESM1_ESM.docx]

**Table S1. Sequence for markers and/or primers linked to white rust resistance in *B. juncea*.**

| **Resistance Loci** | **Markers required** | **Marker or primer (Forward or Reverse)** | **Marker/ gene/ primer sequence** | **Reference** |
| --- | --- | --- | --- | --- |
| *Ac2(t)* | OPB06_1000_ | Decamer primer | TGCTCTGCCC | Mukherjee *et al*. 2001 |
|  | OPN01_1000_ | Decamer primer | CTCACGTTGG |  |
|  | OPB06_1000_ | Forward | GCTCACTTCAGCAGGGGAAGGC | Varshney *et al.* 2004 |
|  |  | Reverse | GGAACCGAACAGACAGACATGAGTTG |  |
|  | E-ACC/M-CAA_350_ | E-ACC (Forward) | GACTGCGTACCAATTCACC |  |
|  |  | M-CAA (Reverse) | GATGAGTCCTGAGTAACAA |  |
| *Acr* (= *Ac2_1_* (*Ac2A_1_*)) | WR2 | Decamer primer | AGGCAGACCT | Prabhu *et al*. 1998 |
|  | WR3 | Decamer primer | AATAACCGCC |  |
| AcB1-A5.1 | At2g34510 | *A. thaliana* gene corresponding to ILP marker | ACGACCACCACGCGGTTTGTGATGAAGAGGGTCCCAAAATGGATACACTTCTCTACCTATTTTTTAACATATCCTACAAAATCTCAATGGTTTTTATTTAGTTCGATAAAGTATTATTCCGAATGTAAATAGTGGAACAAAAAGAACAGCTGGGAGGATTTTGTTTAAAAGGACACTACACTTCCCAGCTTTCCCTCACTGCCCGAAAAGAACACTGCAGAAACACTTTCCTCTGTTCACAGAGAAAGAGAGAGATGATGCTTTACAGCAACAATAGTTGGAGATCGAATTCCATTTTAATACTTCTACTTGGTCTCTCCATTGTGGCCGCCGCAGACTCCGCCGGCAAAACTTCACCGGTCGAAGACGGTACGCTGTTCACTAACATAATCTCATCACTTTTCTTGGTTTTGTTTTTGGGGGGTATAGCTCATAGTGTTAATGGGTTGACGAAAGCATGATTCTTTTTGCTTCTGTGGTTATTTGATTTCGGAAACCTGACCCATTCTCTCTGTTCTCTCTGTTCCAAGAACAGAGGATTCACCATGGCCTGTTGTACTTAGTTCTTCTTTCTAAAACGGTTTACTTTCAGATCTGTATAACACAAAAAATTGGATAAGAAGGAATTTGAAGTTATAATATCCACTTCGATTAGAAAGTTGGGTTCTAGGGTTTATCCTCAATCTCACTTCTTCTTAGTCCTCTTGTTGCTCAGCACTAAGACTTTTTAAGTCGGCTTGTAATCTTGGGTTTAAATTCATGACTGTCTCAGCTGAGCAATGACTAAAAAAGTTTAACCTCATTAATCCTAGGTTTAAAATATGCTAATTCCAATCTAATCTGTCAGACTGTCACAAATCAGAAAGAAAACAAAAGTTTTTGGATATTTTCGTTAATTTAGTATAGTTGTAGGCCATCCTGGGTCAAAGAATAGAAGAGCCCTCCTCAAGTCCTTGAGAGACAGTTGGATTCAATTCAAGTCTTCAAGTTATAAACTCATCTATTTTAAAAATTTGTCTTTAACTCTTTAGACATCTGAATTAGAATAGCATTAACCCCTTAAAAGTTGTAGTTTAACCGTTCCCGCCATGTGCATTCTTTTGTTGTTAGTTTATTTCTTACAAATACAAAACCCTAATTGTCTCCTTTCTTTTGATAAACTAAAATGAATTAAAATCAATTGTAACTAAAATGTATCTTCCCTAAAGAAAAGGGCACTTGTAACTGTAGAGTGTAGACACAAGACCACAAAATGTAATATGTCACGAGTTAATGCTTGGGACCGAGAGAATAAATGGTTCAATTCTCGTGTTTCGCCTTCACTACTTTTGCTATTTTTAAAAAAATTGGTTTTATCGTATTTTTTTCTCCTTTTGTGTACAAATAATTTAATTACCGTAGTGGCTGATAAATGTGAGCTCACGAGAAACGCGAGAATTGAATGAATGTATTTTGGGATGATATTGTTTGTCTAGATTACAAAATCTTGAAAGAATGAATGGCCCAAAAATGGACGTAAAAGATGAAAGATCTATTGTTGTTATTAAGAGACCAACTAATTGTTATACGTCTCCATCCTAGTATTTTTATTTTCTGACACCATTTTCTTAAATCCCGTAATTATTCCCTTTTTCTCAATAAATTACAACTAACTTTCTTGTTCTGCCGGAATCTTTTTTCAAGGCTTGGTGGTTAACGGCGACTTTGAGACACCGCCGTCAAACGGCTTCCCTGATGACGCAATCATCGAGGACACCTCCGAGATCCCGAGCTGGCGATCCGATGGTACGGTGGAGCTAATCAAGTCCGGTCAAAAACAAGGCGGGATGATCTTGATCGTGCCTGAGGGCCGTCACGCTGTTAGATTAGGAAACGATGCAGAGATCAGCCAAGAACTTACGGTGGAGAAAGGCTCTATCTACTCCGTCACGTTCAGTGCAGCCCGCACATGTGCACAACTCGAGTCGCTGAATGTTTCGGTAGCTTCTTCTGATGAACCTATCGCATCGCAAACCATTGACTTGCAAACGGTGTACAGCGTTCAAGGATGGGATCCATATGCATGGGCGTTTGAAGCGGTTGTGGATCGCGTCCGGTTGGTTTTTAAGAACCCTGGCATGGAGGATGATCCTACTTGTGGACCTATCATTGACGACATTGCCGTTAAGAAGCTCTTTACTCCTGATAAACCCAAAGGTAACGTTATATTTAGGTTTTCGATACCAAAGTTTTATAAATCATCCAATAGAAGAGATTTAGAGTAAAGTTAAAAGTAAAATTTTGTACTTTTTACAGGCAATGCAGTGATTAATGGAGATTTTGAAGAAGGTCCATGGATGTTTAGGAACACTACCTTAGGTGTTCTGCTTCCAACAAACCTCGATGAAGAAATATCGTCTCTTCCTGGATGGACCGTCGAATCGAACCGAGCAGTACGGTTTATTGACTCAGACCATTTCTCGGTCCCCGAGGGGAAGCGAGCTTTGGAACTTTTATCGGGCAAAGAAGGCATAATTTCTCAAATGGTTGAGACAAAGGCGAACATTCCGTACAAGATGTCTTTCTCTTTGGGACACGCAGGGGACAAGTGTAAGGAACCTTTGGCTGTAATGGCTTTTGCTGGAGATCAAGCACAGAACTTTCATTATATGGCGCAAGCAAACTCGAGTTTCGAAAGATCGGAGTTGAACTTCACTGCGAAAGCTGAACGTACGAGGATCGCCTTCTACAGCATTTATTACAATACGAGGACGGACGATATGACTTCATTGTGTGGACCTGTGATTGATGACGTTAAGGTTTGGTTCTCCGGGTCTAGTAGAATTGGATTTAGTTTTCCGCTTTTTATTCTTCTTTCTTTGGTTTTCATCTAGATTGTTCCGGTTCAGAAATTGTATTGGTAGACCGGGAATTAAGACGGGATTCCACGTTGTATGATGTATCGTTGTATGATGGATCGGTTCAAGGTACAAGAAATGACAAAGTACTTATGCAATTCATGAGTTTAGAGCAATCATGCTTAAACAATTATCAAACTAGCTAGGCTATACATTTTTGTAGCTTTATTGCAAATTAATGTAAACTAGTTCTTGG | Panjabi-Massand *et al.* 2010, Singh *et al*. 2015 |
|  | At2g36360 | *A. thaliana* gene corresponding to ILP marker | CTAGTTCTTTCGATCTGCAAAATTGACTCTGCGGGAAGAACAAGTCTACTTGAAGCGAAACCAAACGGTGGTGATCCGTGAGATTTCGTCATCATCGGCTTAAAATGCATCACTGGGTTCAAGCTTCTTCTTCCGATTTCAGTGGAACTCCTCCGCAAGCTCGGAGGTATAATTCTATTGTCCTTAGCTCTCGATTTTTGAATTCAGCTGATTTTTTTGGTGTTTGTGTATCAGTGGACATACAGCTGTCAATGTCGGAAAATCCATGGTGGTGGTGTTCGGTGGTCTCGTCGATAAGAAATTCCTCAGCGATATAATTGTTTATGATATTGGTATCATCATCATCATCCTCTGATACCTTTATCATATAATTGTTTTAATTGCGATATACTTGAAGATGAATTAGATGGATATCTTGAGTATCTGTAGAAATGGTTTCTTTCTGGTCTTCGTCGTTGATCATATGTGAAAACTGGTTGTCTTATGATTGTTTGTTTTTTACACTGTCGACTAGAAAACAAACTCTGGTTTGAGCCAGAATGTACTGGCAGCGAGTCTGAAGGACAAGTGGGTCCTACACCTCGTGCGTTTCATGTTGCTATTACAATCGATTGTCATATGTTCATCTTTGGTGGACGTTCTGGTGGCAAGAGGTATGCACTGGTATATTATTTAGTTATAGCGGCTTTACATTCTATTTTCACCGGTCTTTACTAGCTTGTTTCTATTAATCAATTTCAGGTTGGGTGACTTCTGGGTTCTAGATACAGGTACGTATGTAAGTTTTAACAATGTCGTGTGAGACCTGCCTTGGATTTATTAATACTCTGCTTTGGCACTTCTATGATATCTCTATTCATGACAGATGCTAACATTTGCTGCAGATATATGGCAGTGGTCTGAATTGACTAGCTTCGGCGACTTACCTACACCTCGTGATTTTGCTGCCGCTGCTGCTATTGGGAGCCAAAAAATTGTGTTGTATGTATCAAACTTTAGACGTAATCATCTTCTTAGGTTTAAGTTTAAGCTTGCCTGCCTGCATTACTAAACCTAGATCGTTGGCAGGTGCGGCGGCTGGGACGGTAAAAAGTGGCTGTCAGATGTTTATGTTATGGACACAAGTAAAACTTCATTTCTCTATTCTAGTTTTAGTTCTGCAATAAACGGTTTTTATTTGCTGATAATGCTTACTCTCTTGGTATCCATTAGTGTCCCTTGAATGGCTGGAGCTATCGGTTTCAGGGTCATTGCCACCTCCTAGATGTGGTCATACGGCCACAATGGTGGAGAAACGATTACTTGTTTTCGGTGGCCGAGGTAGGCTACACCAAAACTTTGAAAATTCTTGCTGCTGGCTTCTGAATTCACCTTGATTCATTCCTTAAATATGTTTAGGTGTTTGGTATATTCAGTACATAGAAAATATTGTTACTCAAGTTTTTTTTCCCTTTATTTTACTGAACACTGGAACTAATAAGTGTGATTGCTCAAGAGCAGACTCCTGATAGATATAAAAGTCAATGTCAAATGTTACTTTGCTATTCCGCCTTTTCATTCATCAAACAGTGTTTAGGATGTCGATGTGTTTGATGTTCCCATCGCTGACAATTACCAACTTCATTTTTAATGAAGTTTCCTGAATGGGTGTATTGATGATATATGTATTGTTAAAACTCTCGTCCAGTCCTAAGGATTAGGTCATGATCACAGTTTGTTTGGCTTGAGTCAATAACAACAAAACTGAATATATTGTGTGATATGGTTATCTTTGGCAGGAGGTGGTGGCCCAATCATGGGTGATTTGTGGGCTTTGAAGGGTCTGATAGATGAAGGTTCATCCTTAATTTCTTAAAAATGTATTGAATCTAGTAGGTTACCTCTTAATTTGTCTACTTTTCTGTTTATTGATCATATTGTGGTTTACCTTTATTGTGGAAGAAAGAGCGTGAAACACCTGGATGGACCCAACTCAAGCTTCCTGGACAAGCACCATCATCTCGATGTGGTCATACAGTTACATCTGGAGGACATTATGTATGTGTGTTCAGCTTTTGTTTGCTCCGTGTTATTTCTGTAATCAAGATGCTATTCATATGTTTTTGCTTTCGAGTATGGATGGGTTGATGCGACCTACATCACTCACATCATTGAATTTGTTTATGCAGCTTCTGTTATTTGGGGGACATGGAACTGGTGGATGGCTGAGTCGTTATGATGTTTACTATAATGATACTATAATTTTGGACAGAGGTAGGTTTTTGCTCACTCTTCTCATGAGATTGTTCTAGGATCAAGAATTTTTTTACAACTAATGAAATGATTAACTGTGATGTAATTTATCATTTTTCTCGCTGTGCTTTTTTCAATTCTTTGTTAATGTTTCTGCTAAATTTTTGTCTTCACATTCCAGTAACTGCTCAGTGGAAGCGCTTACCAATAGGCAATGAGCCTCCTCCTCCTCGAGCTTACCATACCATGACTTGTATTGGAGCTCGTCATCTTTTAATTGGTGGTTTTGATGGAAAATTGACCTTCGGTGATCTCTGGTGGTTAGTTCCCGAAGGTATTTCATTCTTAATTGTACTAATGCTTTACCTCAAAAAACTTACCAACTAACTTATCATTGTGTGTACACACATCATCATTATTATCTTTGGATATATTTTATATGTTAATATTTTTTAGTGTCTCCAGATAAACTAATCATCCTGTTGGTCATATATTGTAGGCAATTAGCTACTCTTCAATTTCTAGCACGCTCATTTTCTTTCTCTAGTTTGTATTCAAAGTTATGAAATTTACGCATCTGATTGTATTTTTGTGAACTGTTTAGCCGTCTGAAAATTTCTTATTTGTGTAACTGTGTTTAAAGAAATAAATTTATGTGGAACTCTAGATAGAGACCTAACTCAAAACGTTTCCACTTTCACCACAACCTCACCCCATTCTTTTTTGCTTTTCTCAGATGATCCGATAGCCAAGAGGTCTTCTGTGCCTCAAGTTGTAAACCCTCCTGAAATTAAAGAGTCAGAGAGAGAGTTGGACAAGGTGATTGGAGATCCTACATATCATCTTATGTGTTTCTTGCCAATGCAGTCAAAATAGCTTTTGAACTTGTTTAAACAGTTGAGAGTTTGGTCACGGCAGGAAAGGGGACAAGATGGATTCAGTATTGTTGACTTGCAACAGAAAATGGGAATATCTGTTTCATCAGGGTTGCGCCTTCAAATTCCCGAGGAGTCAGAAGATCAAGAGTTTGTTGAATTAGGAACAAGATTAATTGAGGGAGATGTAGTTGATGAACGGGCCTCTATGATTCAGGTGATCTTTCCCTTCTCTATTTCAGTATGTATGATTGTGCTATCCTGTACTAAAATGCTTCCCAATGGAAAGATGGCCGCTCAAGCACTTCGTCAGCATTGGAAACAGTCCACTCCGAGAACTTTACAACTAAAAGAGCTTGGTTCCTTACTTCGAGATTATCAGCGACTTGTTACACGAATTTTCACGTGAGTTCTTTCTTTCAAAACTATCGAAACTGATTCTTTTCTTAAGGTTGTAAAAGTTTTGTTTTCTACGTCTTCTAAAAGCTATTACATGTTATGAAGTTTTACATTTGCTTCTTCCTTTCCTTCACACATGTTTGTTTTCATCGTTGTGGCGAGTGTCTCAGTACACAGAGCAGCTTAACATCTGCTGATTTTGGCCTCCCTGGAACGAAGACCTTTACGTTTTATCATATCAAAAGTTCTTCCGAGGTAATACTGCATGATTTCCTCCTGTTATTACCTCTCTTGAACATTTGTAACCAGTTAATCTATGTGTTGCATCATCGTAAGGACCCTTGTACGGATTTTACCTTGAATTATGTGGCTGTCTAAATAATCTATTTTTAAGTTGATTTGAGCACATGCACTCAGAATACCCAATACACTGGCCCAAAAGAAATAGATGTTACCACTGTGATGCATAACAGCATTGACGATATGCATTACTCTTATCTCTTCCTTATTTTTCCGTCTAAAACTTTTATGCATCTTAACACACACATCGTTTTTATGATTTGCACATGCACTCTGACATGTCTAAGTCCATTGATATTCTCAGTTGCGGATTAATGACATCTCGAAGTTGCTGGAAGAATACAAAACTCTTGTAATCTGATTGAGGAGTCGCCACAAAACTCACGAGGAACAAAAATATCTTTAGGTA |  |
| AcB1-A4.1 | At5g41560 | Forward primer | CCTCACAATTTCAGTCAACATCGT | Panjabi-Massand *et al.* 2010, Singh *et al*. 2015 |
|  |  | Reverse primer | GAGGTGGAAGAGTACGGTTGTG |  |
|  |  | *A. thaliana* gene corresponding to ILP marker | AGAGAAACATTAATTTGAAGCTGGTTCTAGAGGTAGGCCCAATGGGCCCATAAACCCTTTTTCGTTTCTCCCTTTTATATCTCTCGTCTCTCTCTCTCTCTGTCTGAAGAGAAGGAAAGATCATCAATCACGATTCCAATGGCGTCGATTCTGGGTGATTTGCCTTCCTTTGATCCTCACAATTTCAGTCAACATCGTCCCTCCGATCCTTCTAATCCCTCTGTTAGTTTCTTCCCCCAAATTCAATTTTTCAATTTTACGGATCTGAGTTAGGCTTTACTTGGTCGTATTGGAAAAAAAATGTGTCCTTTGTTGATTCAAAGAGATGTAATTCGAATGTGTATCTGGGTTTTGCTGCTTACTTGGTCAGTTCCAAAAAGTTCCATCTTTTCAATTATCATCGAGTTTGCTGTTGGATCTTGTGAAAAACCAATACAAATTAGCCATTTTTGTCAGATTGATTGATCTTAGAATCATAATTCTGATTCCATTTGGCCATAATTTAGCTGCTAAGTGACGAAGACAAGTTTCAACTAGCTTGTATCAGTTAAAGATTAGAGTTTTGATCTGTTATCGAAGGTTTGAGTTTTTTGTTCATGTTTTCTTGCAGAAGATGGTTCCTACCACCTATCGTCCTACTCACAACCGTACACTTCCACCACCAGATCAAGGTGAAACAAAAAATCGTGCTTTTTTGAAAAACCTTGCGTGTTTTTTCGGCTAGAGATTTTAGAATTTCGTTACATTTTATATATAGTGTCAAAGATTTGTCTTATGAAGTTGTGATCTTGAACTTGCTTTGATTGAGTGATTTAGTTACTGTCTTTACTATGTATCACTTCTTAGAATCTCTAGGCAAATTGGTGTTAATCAGATTCAACAGTCTCGAGTTTTCACAGATCATGTCTTATGTTTTTACTAATTTGTATCTTGTTCGTTATGGTTGTAGTGATAACTACAGAAGTGAAAAACATTCTTATACGCAGCTTCTATCAACGAGCTGAAGAAAAGGTGAAACAGCTCTCAACTCTCATTCCTCATAGTTTGTGATTCTTTGATTCAAATCTCCGTTGTTTCCCTCGTATATATAGTTCATAACACAGGATTTCGTAGGAAAATAGACAAAAGAAAACGATATAGAACAATCTTGAACTTCTTCAGATAACAAAACTGTTGATTTTGGTTGTGTTATCCGAAATCTTAATTTGTTTTGTCAAATTTGTCAATTGCAGTTAAGACCAAAGAGACCGGCTACAGATCATCTGGCAGCGGAGCACGTGAACAAGCATTTCCGTGCTGCGTCTTCTTCTTCATCTACTCAGGGTTTATAAAAAACTTAAGTTCAAGCCTATAACAATGGTCATTTGTATGAGTACCTCTTATTAGTGTTTTCAATGTAGAAAAAAAAAGATAAGAGCAGTTCATGAGAGAAATGTAGTGAAAATGTGTGTGTACATAACATTAATGTTTCTTTTATTTCTTGACTTAAAGTTGCTCACTATTCACTAGCGTTGAACATGAAAATCAGGTTACGTTAACGAAGTTACGAGCCCAATATCAA |  |
|  | At5g41940 | *A. thaliana* gene corresponding to ILP marker | GAAAGAAGAAAGAATGACCCAACTGTATGAAGGCCCATTAAAAAAGTGGGCCCAATTTCTATAAACGACCTTCGACTTTTTCAATAATCAATTTTCATCCAATTCAACTCAGGAAATTGCTATGATTTTACTCTGAGAATCAGAAGAAGAAATCTCGGTGAGCAAAATCCCTAGTTTTGGGATGATGTTTCCACGACTACGATTGACGAAGGTCTTCTTCTCCCCGATCATCGCCGGGAAATGGATCGTCTTTGCCGACGGTAGCAGCGGCGGCGGCGTTGGTAGAAATGGCTACTACGGTGGATTGTGGTCGTCGGCCGTTATTCCTTCCAATGTTGGTTTAGCTGTCGCTGTCACCGTCATGGCCGTGGCCGTCGCTTTCACCGTCTACTCTCGTAGGTATACACACAAAAACGATCTGATTTATGTTCTAATCGATTTCTTCAAAGCAAAGAAGCAATGCTTGTTCACTACTTAGTTAAGTAGTTATTTGGTATCCATAGATTTGATTGGTTCTTGCTTCTTTGTTACCTCACCAATTCGAGGCTGGATCGTCATGCCTTTAGGTTCGAAGAATTCTCTGTTAGTTAGGTTATTGTGATTGAGTTCTTGGTCCAATGTTAGCTGCTAACTCGTACTTCCACTACCTCTTCTTGTCATGGAACTTGTAAGACTCTCAGGCTGAACATAGTCCTGGCCATATTCTGTATTCATAGACTCTGATGCGTATAATGATTTTGAGGCTCGAAGCACTAAGGCTTTTTATAGTATAATGAGGGAAAATGTAACCAATGTTTAATGTTTACTTTTAGGTTGTTTGCTGGGTTGTCTCTTTTTGTATGACATGCTCTCTTGGTTAGTATTGAACAAACAAAGTGATTCTAGAAGGGTCCTTAGGTGGAAGTCTGAAACTTCATGAACTTTGTATAAACTCCTAAAGGAACTCTCGATAATCAACTCCCCAAGCTTTGTATTTGTGTTTCTTCTTTTGCAATTTTGTCAGATACATAAGCTTTTTGTTGCTAAATCTCTTATCATCGAATTCAGAAATCTGTTTTTGTTGTTATAACTCTTTCTACAACCCAAGCAATGAAGCTATTTTGTTTAAGTTCTGCATCATAAATATGCATACCATGAAGAGTTGGATCGAATAATTGCAGCCTGATATGATCTTTTGTCTTCTGGTCCTTATTCTAATTCTTGGCATCTCTTGGACTGTTTCTGTATGTAGTGATGAAGTGGTTAAGAGTCTTAGGTCTTCTGATGTGTGATAGAACTCCATTGATACTTTCAATTATTTTTAACATATATGAATATAGGACATAACATGTACCATTTAGCCTGTCTTATTTGTTCAAAGAAAGCTTTTTATCACTACATTTGGTTTGGTTTTATGTAATTTTTTTTCTTTCTAAACTTACTGGACGAAGTTGCTCATTTTCTGCTCATCTACTTCTTCAGAGGAAGCATTGGATCACCGTGGTCACTAAGGAGAAGAAAGCGAGTTCTTCAACCTAAACAATGGAATGCTTTTTTCACAGAGGAAGGGCGACTCAGTGATGGAGGTGTCAAATTTCTGAAGAAAGTTCGTAGTGGGGTATGTTGCTAAATAAGAAAAAAATCTGCTGTAATCTGCAGTTGATAACCTTTTGACACTTTGATATACTAATTTTTATTTTTGTTTCAGGGTGTACATCCAAGCATCAGACCAGAAGTTTGGCCGTTCCTTCTTGGAGTGTAAGAATCAGCATCTTGTCACTTTTCCTTGTTTTTTGTATACTTTTATGAGTTTTATCTTTTTCGGCTACCATTGGGGGTTTTCTGTTCGGCCTTATGTTCATACTCATGCATGGTCTATTGATATTAGCTTTTTCTTTGGGGATATTGATGTTATGTAGGTTTAAGGTTGAATAAACAGTGCATCAGTAAAGCATTCATTAACTAGGAGATCAGAGAGTTGCCTTAATGTTTTTCTATCAAGAGATTTATAAGATTAGTCCTAGTTTCGCCATACCAAACTCCACCAGCCGTCGCCCTACATTCCATTAACTTGATAATGAAACCTGTGCAAGTTATTTGTAGTCTTTATCATCTCGACACATTGTAACCTTATGCTACTTACCTCTCACAGGTATGACTTAAAGAGCAACAAAGAAGAAAGAGACTCCATTCGACAGCTGAAACTGTATGTTTGCTTATACTTATTGAGAGTTTAATGGCTTCTCAACTTTCTACCAAATGTTACGTTTCCCATTTATCTCTGTTCTCCTAGGTGGCAGCTGGTTATTAACTTGCTCAATGTGTTGATAAAACTAGGAATAAGAAGTAGTAGTTTGGAACTCCTACACTGAGATTTCGAGTCAGTTTGTAAGTAACGTCTTTTATATCTGTAAATCCTGCAGGACGGAATATGAAAACCTGCGGAGACAGTGTCGCGAGATTCATGAACGCTATGAAAATGTCTGCGACTCAAAGGAGACAGCTCAGAGCAGCAACACTGAAGACAGTCAGGTTCTTGAATCCCATGATATTGAGGAAGTCGAGAGTTCCACAAGAACCATCACCGTAGATGAATCAGAGAAGTTGAATTCTGAATCGATTATGCAGGATGAGAACTGTGAAAAGAGCGATGTCACCACAGAAGATGCTGCTGGTAATGATTCAGACTCAACCAATCCTGAAGAAACCGAGACTTCACCTTTTCTAGCTAACGAAGAAGTAGAAAGCCACAATACTGTTAACCAAGAAAAAGAAATTTCGTCTCCATCATCAAAGCCAAAATCTCAGGCAGCGGACGAGGATTTCATGTCAACTTGGCAGAGAATTATCCGCCTAGATGCTGTGAGAGCAAATGACGAATGGGTGCCCTACTCACCAACTCAAGCTGCTGTTTCTGAGACAAAAGCCCGGGGAATAGCCATTCAGGTTGGTCTCAATGACTATGACCACTTGGAACCCTGCCGGATATTTCACGCAGCCCGCCTGGTCGGGATCCTTGAAGCCTACGCAGTCTATGATCCAGAAATTGGTTACTGTCAAGGAATGAGCGACTTACTATCTCCTTTAATCGCAGTGATGGAAGACGACGTCTTAGCATTCTGGTGCTTTGTTGGCTTCATGAGTAAAGCACGGCATAATTTCCGGTTAGACGAGGTTGGAATCAGGCGGCAACTCTCAATGGTATCGAAAATCATAAAATTCAAAGACATTCACTTGTACCGACACTTGGAGAACCTTGAAGCAGAAGACTGCTTCTTTGTATACCGTATGGTTGTAGTTCTGTTCAGACGAGAACTAACCTTTGAGCAGACGTTGTGTCTCTGGGAAGTAATGTGGGCGGATCAAGCTGCAATACGAACTGGGATTGCGAAGGCGACATGGGGGAGAATCCGGTTACGAGCACCACCGACAGAAGATTTGTTGCTTTACGCGATAGCTGCAAGCGTGCTGCAGAGGAGGAAGACGATAATAGAGAAATACAGTGGGATGGATGAGATAATGAAGGAATGTAACAGCATGGCTGGTCATCTTGATGTTTGGAAACTTCTAGACGATGCTCATGACTTAGTCGTCAATCTTCACGACAAGATCTAAGACTACTTATATCTTTCACTCTTGTTCTCTCTCTTTACTTGTTTGTTGCATTCTTTGTTATTTGTTACATAAAATGTGATAAAAACCGTTGTCCTAAAACGCATGAAAATGTCTTCACTATTGCTAAAATTAAATGTAGAAAAACTTAGCCAAACGAG |  |
