# Supplementary material for: In silico integration of disease resistance QTL, genes and markers with the Brassica juncea physical map
Source: Mol Breed. 2022 Jun 27;42(7):37. doi: 10.1007/s11032-022-01309-5 (PMC10248627; doi:10.1007/s11032-022-01309-5)
Supplement: Supplementary file 2 — Supplementary file2 (DOCX 26 KB) [file 11032_2022_1309_MOESM2_ESM.docx]

**Table S2. Sequence for markers and /or primers linked to blackleg resistance in *B. juncea*.**

| **Resistance Loci** | **Markers required** | **Marker or primer (Forward or Reverse)** | **Marker/ gene/ primer sequence** | **Reference** |
| --- | --- | --- | --- | --- |
| *Rlm6* (= *Jlm1*) | OPG02.800 | Decamer primer | GGCACTGAGG | Chèvre *et al*. 1997 |
|  | OPT01.760 | Decamer primer | GGGCCACTCA |  |
|  | OPI01-HaeIII | Decamer primer | ACCTGGACAC |  |
|  | B5-1520 | Forward | TGCCTTTCTCACTTCTTCTCTC | Rashid *et al.* 2018 |
|  |  | Reverse | AGCGTCTATGTCGGTCTTTCAA |  |
|  | B5Rlm6_1 | Forward | GTTACAGAGGGTTGTATCTCATTC |  |
|  |  | Reverse | ACCAGGAGTGGTTAGAAGCTAAT |  |
|  | BjHZ-1 | Forward | CCAACCCTTCGAGGTCAATA |  |
|  |  | Reverse | CCAGAGACCCCAGTTAAGCA |  |
|  | BnHZ_2 | Forward | TCCATGATGTGATAACTATAGACG |  |
|  |  | Reverse | TTAAAGTTTGTGAATTTCTTCCTT |  |
| *LMJR1* | PN199RV | RFLP | TAAGTTAAACATACAAACAATAAACGAAAGCCAGATTGAAAATACTAATGGTACTCACTTTCTGGATCATGCTCCTTCGCAATCTTCGGAGCCCTGGCTAGCAGGGACTATAATCAGCAGTATGGCATCATTCAGCAATCTTGCATCCAAAAACAAACAAAAGTTTCAATAAGGAGTCAAGGACAAACATGATAATGAATATATCTTAGAATTACAGCAGGAAATCCGATTAACATTGTTTAAATCTTATGTTCATGGGCATGAAAAGGCACTATACAGGCCCAATGCTCACCATTGAATCATCCACAATTCTTTGGTCCAGTCCAGGCAGGTCAACTAATTTCAGTGGTGGAGCTGAAATATAAAGGGTAAAAGCAATATGTTATGGTGATCAAATAAAATTTAACACTAAAACAATGCAGGTCATATGTCACACTATTCACGTTCAAAAGAGTACTATATATTTGAAGCTCCATTATACATATTATATGATATCCATCAACTTAAAGCTTTAAGCTCTAAAAAGTGCAATATATCGTATCGGAGAGGGGGATAGCAACTTATTAATGCTGGCTAGTTCACTGATATCAAAGATATATTCAATCATGCAATTTACATTTGCTGAGGATGTGCTACTATAAGAGTAGATAGGCATGCCACTAATAACTGTGCAATTAAAAAAAGGGCTTACCTGTGCTGGTACGAAGGTTTAAATATATTTGATCATGATTCTTCCCTGAGGCTCCTTTGCTAAGTCTATCTGGAAGAGAGTGACTCAGAGCACTTATAACAAACCCATGTAGAACACAATGGTAACACTATGAGAAGATTGATCAAATGCATTATATTGTGCATGCAGAAAGGGCCTCACACACCACTGCCCAATTACATATGTTGATAGAAGGAGACTTACTTGCTGAAACTTGTTGAGATTTGTTGTCAGTTTGCAAGATAACAGCTTCTCAACGAAAGTCAATTATTATAGGAGCTCGTGTAGCACCATTGTCACCTGTAGGCTGTCCACAGCAAACAGAACAGACGTGAGGATTATTTGATAGAAATGAACGATCCAATAATAAACATAAGAAACTCCCACAAGACTCAACGTACTAC | Christianson *et al*. 2006 |
|  | sJ3627R | SSR | ATCTCAGCTTGCCATGGTTT |  |
|  | sB1822 | SSR | TCGTTTATCCCGCGTTTATC |  |
|  | sB1672 | SSR | GAACGGAAGGTTTTCAAACAA |  |
|  | sJ7046 | SSR | GTTTGCCGTCTTCCAGATTT |  |
| *LMJR2* | PN120cRI | RFLP | CTGGTTATTCGGTTTATGTGGGCCAAGATGCATATAGAGAACGGGGACATAATGAGGGATCATTGATCCAGCCCTCGCGGAAGACTATTCCTGTCTCCAGTCAATGCGGAAGATTGCAGAGAAGGCGTTGCTGTGTGTGAGACCTCACGGGAACATGAGACCGTCTATGTCTGAGGTGCAGAAAGATATTCAAGACGCGATAAGGATTGAGAAGGAAGCTTTGGCGGCCACAGGAGGCTTATCAGATGATTTTTCTAGGACCTCGGGGCATTCGTCGTCTCTCAACATGGGAATGCATGACTTGGCTGGTTCGCACAACTTTGTGGCTATTGATGAGTCTGTGTTGCAGCCAAAAGCTAGGTAGTCTCAGGATTCAGCTTTTGTACACATGCATCACTTTCACTAGAGTCCTTTCTTTAACCTTTGACTTGCTTTTCTATACTATTATTCTGTTTAGGCACGTGGGTTCAGGTAGTTTGGTTCGATCATAATCTTTTGCCGAATTGAACCGGAAAAGTTTTTAAAAAATTTATTGAAACTAACCAAAGTTTTTGGTTTTGATTATATTTTCGGGTTGAAAATTTTGTTTAAATTGATTAAATTTGAATAATTTAAGTTGGTTCAGTCAAAAAATTGGTTAATTAGATTCGTTTGATCCCAATTTTTTATTTTCGAAAACTGAACTGACTGATTATCAAACTGAAAACAAAGTTTTTTATAAACCTGTCGAATTGGACCGAACTCGTTGTTGTGTTCCAAGTAAACAACAAAGTTCAGCTTCATAAATGTTGATCAGAGTTTGAGCTTAGTCACATAGACAAAGAAGCTAAAGCAGTTCAGAATACAGCTTAGGGAACTAAAACATGTCTACACATTGAAACTTAAAGAAAAAACAACAAGCGAAAATTAAAGACAACAATGGTAGATTTTTTTTTTTAATCTTACTTCAGTTATCACTTCTGACTCTTCTTAAGTAACACACCAAACTTCTTAAGCATACTCCCATTCTTCTTCTTCGGCGAAGACAACAAGTCATCATCAGTTTCATCTCTATAAGGCGACAACATGTTCACGTTCCTCCTCAACGGACTCTCCAGCGATCCTGTTCTCTCCACATACTTCCCATTACTGTTATTATTACCACCAGAGAGCATAGAAGCTGCA | Christianson *et al*. 2006 |
|  | sB1728 | SSR | TGTTTCTCGATCTGGCACTG |  |
|  | sB1668I | SSR | GATAAAATCGGGCTCGACAA |  |
|  | sJ34121 | SSR | TGCTTAAGTCCATCAATGCG |  |
|  | sB5162 | SSR | CACGGCTCTCAACTCTTTTG |  |
|  | sB3739 | SSR | AGCAAATTTATGGTGGGCAG |  |
| *r_j_lm2* | B5-1520 | Forward | TGCCTTTCTCACTTCTTCTCTC | Saal *et al.* 2004, Saal and Struss 2005 |
|  |  | Reverse | AGCGTCTATGTCGGTCTTTCAA |  |
|  | C5-1000 | Forward | GTGGAAGAAGAGTTAGGGATAGAGC |  |
|  |  | Reverse | TTGGATAGAGAAAATGGAAGTTGTT |  |
|  | RGALm | SCAR (RGA-derived) | GTTCGAAAACAGAGCGGTGATTTCGGAAGAGCTTTCAAAACAACTTGTCAAGAAAAAACAGAGGAAGTGAAGCAGAGATGGACAAAGGCTTTGACTGATGTCGCTGACATAGCCGGAGAACACTATCTTAACTGGTTTGTCTTTTTTTCTTTTCTTTTGGCTCAGTGAATTATAATGCTTTGATCTTTCTGACCAAAATGGCTCATCAAATTGGCATGATTTTGATTTAAAAAAAAAAAGCCACCGAAAAGAATTTTGTTTCTTTGATTTATTAGGGACGATGAAGGGG |  |
|  |  | Forward | TTCGAAAACAGAGCGGTGATTT |  |
|  |  | Reverse | CCCCTTCATCGTCCCTAATAAA |  |
| *PhR2* | S7G4 | STS (AFLP-derived) (Resistant) | TTTAAATGACTTTGGGATTCCAATGCTTAAGATATGAATGAGGCCTTATGTCTAGAGTGACTTGGGTTCCAAGAACCATGCCATGGCGCATTTGATCCTTTCTCCACTCTTTTTTATGAGCAACATCTTTTCTCCACTCTAATAAGTCCACAAAGTGTTAGAAATGATATCATATTTATTTTGGGATATAAATTTGAATGAGTAAAAATGTTTCTTACAATCATATGTGTCATGCGAGAAACACGTGTGTCGTGTGAACTGACTACTCATCCATTACTCAATCCACAACATAAGGTTGTATTCATGCCATTCTTTTAACCGCATATTACGTTTTATCGTGTTATCATATACATCCAAGCATTTTTATATATCCTATCTATAACATGATTAAAGAGAATCTGAGCATAGGACAAAAGATCTGTGACAGAACCATATCTAATCTAATCTTTATGGGTGAGAATCGGACCCAGTTGTCCACCCTACAACTACCGTTTACACATGGTTCATGAAAAACTTGTAAATCAAGTTGTATAAGCACACTAAACTAAGGGTTTATCGGTAGAAGAAATTTCGAGGAATTATAACATTTGAAGATTCTATTGTTTTTGGTTTATGAATTCTTAAAATCTTTTGTTATGGGGCGGAAATTTTTAAA | Plieske and Struss 2001 |
|  |  | F1 (Resistant) | AATGACTTTGGGATTCCAATGC |  |
|  |  | R1 (Resistant) | GTTATGGGGCGGAAATTTTTAA |  |
|  |  | F2 (Resistant) | CTTTTAACCGCATATTACGTTTTATCG |  |
|  |  | R2 (Resistant) | CAAAAGATCTGTGACAGAACCATATCTAA |  |
|  |  | STS (AFLP-derived) (Susceptible) | TTTAAATGACTTTGGGATTCCAATGCTTAAGATATGAATGAGGCCTTACGTCTAGAGTGACTTGGGTTCCAAGAACCATGCCATGGCGCTTTTGATCCTTTCTCCACTCTTTTTTATGAGCAACATCTTTTCTCCACTCTAATAAGTCCACAAAATGTTAGAAATGATATCATATTTATTTTGGGATATAAATTTGAATGAGTAAAAATGTTTCTTACAATCATATGTGTCATGCGAGAAACACGTGTGTCGTGTGAACTGACTACTCATCCATTACTCAATCCACAACATAAGGTTGTATCCATGCCATTCTTTTAACCGCATATTACGTTTTATCGTGTTATCATATACATCCAAGCATTTTTATATCCTATCTATAACATGATTAAAGAGAATCTGAGCATAGGACAAAAGATCTGTGACAGAACCATATCTAATCTAATCTTTATGGGTGAGAATCGGACCCAGTTGTCCACCCTACAACTACCGTTTACACATGGTTCATGAAAAACTTGTAAATCAAGTTGTATAAGCACACTAAACTAAGGGTTTATCGGTAGAAGAAATTTCGAGGAATTATAACATTTGAAGATTCTATTGTTTTTGGTT |  |
|  |  | F1 (Susceptible) | AATGACTTTGGGATTCCAATGC |  |
|  |  | F2 (Susceptible) | CTTTTAACCGCATATTACGTTTTATCG |  |
|  |  | R2 (Susceptible) | CAAAAGATCTGTGACAGAACCATATCTAA |  |
|  | pRP1513 | STS (RFLP-derived) (Resistant) | TGTTACTGATTGTGCAGAACGCCTTGAGGCGCACAATGGAAACTTACTCCAAAGTCACCAGATTCTTTTTCATCTGTAACTATATCAGCAGGTAATTTCTTTACCATGGTTTCTTTCGTTTATAGACTCTCAAGTCTTATGGTTAGAGCTGATAGTTATAAAATTTGTAATTCATAATGAAAAACATTTTGTAGAGCAACTGAAATGTGGACCCTCCGATTTTGATAACCTTACATTTCTTTTTCTGAAGGATCATAGAGCCCCTTGCTTCAAGATGTGCGAAGTTCAGGTTTAAACCACTTTCTGAAGAAGTCATGAGCAACCGTATATTGCATATATGTAATGAAGAAGGTCTCAACCTTGGTGGAGAGGTGTGCACATATGCTTTCAAGGTTTTCCTTTTTATTTATTTAGTCTTTCTGGAAACTACTTATACTAGCTACTTTCCGTGTAGGCTCTTTCAACTCTGAGCTCCATATCACAAGGTGATCTCCGTAGGGCCATCACGGTATCTTCAGGTTAAGACGAAAACTGTCTGTGAAATATAGTTACTGGACGAAATTGACTGTGGTTGTTGA |  |
|  |  | Forward (F1) (Resistant) | TGTTACTGATTGTGCAGAACGCCTTG |  |
|  |  | Reverse (F1) (Resistant) | CTGGACGAAATTGACTGTGGTTGTTGA |  |
|  |  | STS (RFLP-derived) (Susceptible) | TGTTACTGATTGTGCAGAACGCCTTGAGGCGCACAATGGAAACTTACTCCAAAGTCACCAGATTCTTTTTCATCTGTAACTATATCAGCAGGTAATAATCTTTTATTATGCTCCTAATTGTTGGGTTAGATTGTCTTTCGTTCTTTGCACCTGTTCTCCACTTTTTTCTCTTTGGGTATGTTAAATGGTTGCTTCCGTTTATACTATAGACTCACAAGTCTTATGATGAAGCTGCTAGTTGACCATTTTACTTATTCGTAATCAAGAACATTTTGTTTTGAGGGCTGAACTATGGAGCCACCCGATTTTGATAACCCTTCATTTTCTTTTCTCAAGGATCATAGAACCTCTTGCTTCAAGATGTGCAAAGTTCAGGTTCAAACCACTTTCTGAAGAAGTCATGAGTAACCGTATATTGCATATTTGTAATGAAGAAGGTCTCAACCTTGGTGGAGAGGTGTGCACATATGCTTTCAAGGTTTTCCTTTTTATTTATTTAGTCTTTCTGGAAACTACTTATACTAGCTACTTTCCGTGTAGGCTCTTTCAACTCTGAGCTCCATATCACAAGGTGATCTCCGTAGGGCCATCACGGTATCTTCAGGTTAAGACGAAAACTGTCTGTGAAATATAGTTACTGGACGAAATTGACTGTGGTTGTTGA |  |
|  |  | Forward (F1) (Susceptible) | TGTTACTGATTGTGCAGAACGCCTTG |  |
|  |  | Reverse (F1) (Susceptible) | CTGGACGAAATTGACTGTGGTTGTTGA |  |
| Un-named locus | OPU9 | Decamer primer | CCACATCGGT | Struss *et al.* 1996 |
