# Supplementary material for: In silico integration of disease resistance QTL, genes and markers with the Brassica juncea physical map
Source: Mol Breed. 2022 Jun 27;42(7):37. doi: 10.1007/s11032-022-01309-5 (PMC10248627; doi:10.1007/s11032-022-01309-5)
Supplement: Supplementary file 3 — Supplementary file3 (DOCX 21 KB) [file 11032_2022_1309_MOESM3_ESM.docx]

**Table S3. Sequence for markers and /or primers linked to hypocotyl rot resistance in *B. juncea*.**

| **Resistance Loci** | **Markers required** | **Marker or primer (Forward or Reverse)** | **Marker/ gene/ primer sequence** | **Reference** |
| --- | --- | --- | --- | --- |
| *BjCHI1* | Gene | Gene | AACATGAAGACTTATCTCCTTCTCCTTCTCATCTTCTCACTTCTCTTATCATTTTCCTCCGGTGAGCAATGCGGTAGTCAATCCATACCCGAGGGAGCACTCTGCCCCAACGGTCTATGCTGCAGCGAGGCTGGATGGTGCGGCACCACCGAAGCTTACTGCGGGCATGGTTGTCAAAGCCAGTGCAATCCTGGTCCCTATCCTCCTCCTCCAACCCCGCAGTGTGGTCGTCAATCCATACCCGCGGGAGCCCTCTGCCCCAACGGTCTATGCTGTAGCGAGGCTGGATGGTGCGGCACCACCGAAGCTTACTGCGGGCATGGTTGCCAAAGCCAGTGCACTCCCATTCCCACTCCTCCTGCTCCCACTCCCACTCCTCCTACTCCCACTCCTCCTAGTCCTACCCCTCCTGGTCCCACTCCTCCTGGTCCCAGCGGGGATCTTTCTGGCATCATTTCAAGAGATCAGTTCTATAAAATGCTTAAGCACATGAACGACAATGATTGTCATGCTGTTGGTTTCTTCACTTACGACGCCTTCATCACCGCCGCTAAGTCTTTCCCAAGTTTCGGGAACACCGGAGACCTTGCCATGAGGAAGAAGGAGATAGCAGCCTTCTTCGGCCAGACTTCCCACGAAACCACCGGTGGGTGGTCGGGTGCACCCGATGGAGCAAATACATGGGGCTACTGTTACAAGGAAGAAATTGACAAAAGCGATCCCCACTGTGATAGCAACAACCTCGAGTGGCCATGCGCACCAGGCAAATTTTACTACGGACGAGGACCAATGATGCTGTCTTGGAACTATAATTACGGACCGTGCGGGAGAGACCTAGGACTCGAGTTACTCAAGAACCCAGATGTTGCGTCCAGCGACCCAGTGATAGCTTTCAAAACCGCCATTTGGTTCTGGATGACTCCTCAAGCTCCTAAACCCTCGTGCCACGACGTGATCACCGACCAGTGGGAGCCGTCGGCTGCCGACATTTCTGCCGGAAGGTTACCAGGTTATGGAGTGATTACCAATATCATCAACGGTGGATTAGAGTGTGCTGGTCGCGACGTCGCAAAGGTCCAAGATCGGATATCGTTTTATACAAGGTACTGTGGCATGTTTGGTGTTGATCCTGGAAGTAATATTGACTGTGACAATCAAAGGCCGTTTAATGAAGGTAGTAACGTTTTCTTGGATGCTGCAATTTAATAAGTACTGTTAATGAAGCTTTGTTGTATCCAAGCAATAAGAGAGTATCAAATTAAATTAAATAAAACTCCTTTTTATTAAGTAAAAAAAA | Zhao and Chye 1999 |
|  |  | Forward | GGTGGATGGGCTACAGCACCAGAC |  |
|  |  | Reverse | CCACGTCCACACTCCAA |  |
|  | PCR 1 | Forward (P1) | CCTCCGGTGAGCAATGCG |  |
|  |  | Reverse (P2) | TTAGCGGCGGTGATGAAGG |  |
|  | PCR 2 | Forward (P3) | TCCTCCAACCCCGCAGTGT |  |
|  |  | Reverse P2) | TTAGCGGCGGTGATGAAGG |  |
|  | RT-PCR | Reverse-transcription primer (P4) | CCACTCGAGGTTGTTGC |  |
