# Supplementary material for: In silico integration of disease resistance QTL, genes and markers with the Brassica juncea physical map
Source: Mol Breed. 2022 Jun 27;42(7):37. doi: 10.1007/s11032-022-01309-5 (PMC10248627; doi:10.1007/s11032-022-01309-5)
Supplement: Supplementary file 4 — Supplementary file4 (DOCX 17 KB) [file 11032_2022_1309_MOESM4_ESM.docx]

**Table S4: Studies that identified molecular markers linked to white rust (*Albugo candida*) disease resistance in *B. juncea*.** *Arabidopsis thaliana* genes that the ILP markers were based on were utilised to identify the position of the ILP markers.

| **Resistance gene or locus** | **Source of resistance (cultivar, line or accession)** | **Population** | **Markers utilised in study** | **Markers linked/ associated to locus** | **Marker (and/ or primers) sequence publicly available** | **Reference** |
| --- | --- | --- | --- | --- | --- | --- |
| *Ac2(t)* | BEC-144, BEC-286 | F_2_ and F_7_ Recombinant inbred lines (RIL) | RAPD | OPB06_1000_, OPN01_1000_ | Decamer primer only | (Mukherjee et al. 2001) |
|  | BEC-144, BEC-286 | F_2_ and F_7_ Recombinant inbred lines (RIL) | CAPS, AFLP | OPB06_1000_ (CAPS (RAPD-derived)), E-ACC/M-CAA350 (AFLP) | OPB06_1000_ SCAR primers only | (Varshney et al. 2004) |
| *Acr* = *Ac2_1_* (*Ac2A_1_*) | J90-2733 (Donskaja x Jubilejnaja) | F1-derived doubled-haploid (DH) | RAPD | WR2, WR3 | Decamer primer only | (Prabhu et al. 1998) |
|  | J90-2733 (Donskaja x Jubilejnaja) | F1-derived doubled-haploid (DH) | RFLP | X140a (co-segregating), X42, X83 | No | (Cheung et al. 1998) |
| AcB1-A5.1 | Heera, Donskaja-IV; Heera, BEC-144, JMY-11 | Resistant lines crossed with Varuna or TM-4 (susceptible), DH population (VH - Heera; -Donskaja-IV); Resistant lines crossed with NRCDR-02 (susceptible); segregation population (F2) | ILP, AFLPs, SSRs | At2g34510, At2g36360 (ILP marker) | No^1^ | (Panjabi-Massand et al. 2010, Singh et al. 2015) |
| AcB1-A4.1 | Heera, Donskaja-IV; Heera, BEC-144, JMY-11 | Resistant lines crossed with Varuna or TM-4 (susceptible), DH population (VH - Heera; -Donskaja-IV); Resistant lines crossed with NRCDR-02 (susceptible); segregation population (F2) | ILP, AFLPs, SSRs | At5g41560, At5g41940 (ILP marker) | At5g41560 primers only^1^ | (Panjabi-Massand et al. 2010, Singh et al. 2015) |

1. ILP markers are derived from *Arabidopsis thaliana* genes. The corresponding *Arabidopsis thaliana* gene from the markers are available and can be utilised to locate the physical position of the ILP marker on the *B. juncea* reference genome.
