# Supplementary material for: In silico integration of disease resistance QTL, genes and markers with the Brassica juncea physical map
Source: Mol Breed. 2022 Jun 27;42(7):37. doi: 10.1007/s11032-022-01309-5 (PMC10248627; doi:10.1007/s11032-022-01309-5)
Supplement: Supplementary file 5 — Supplementary file5 (DOCX 18 KB) [file 11032_2022_1309_MOESM5_ESM.docx]

**Table S5: Studies that identified molecular markers linked to blackleg (*Leptosphaeria maculans)* disease resistance in *B. juncea*.**

| **Resistance gene or locus** | **Source of resistance (cultivar, line or accession)** | **Population** | **Markers utilised in study** | **Markers linked/associated to locus** | **Marker (and/or primers) sequence publicly available** | **Reference** |
| --- | --- | --- | --- | --- | --- | --- |
| *Rlm6* (same resistance locus as *Jlm1*) | *B. napus* introgression lines (with genetic material from *B. juncea* cv. BJ168), Aurea, Picra, Zaria, Stoke | Resistant line crossed with *B. napus* cv. Samourai (susceptible); segregation populations (S3, B3F1-1, B3F1-2) | RAPD | OPG02.800, OPT-01.800, OPI01-HaeIII | Decamer primer only | Chèvre et al. 1997 |
|  | *B. napus* introgression lines (with genetic material from *B. juncea* cv. BJ168) | Resistant lines crossed with *B. napus* cv. Samourai (susceptible) followed by backcrossed with *B. napus* (susceptible) and/or selfing; segregation populations | RFLP | OPG02.800, OPI01-HaeIII (RAPD-derived RFLP) | Decamer primer only | Barret et al. 1998 |
|  | X | X | SCAR, CAPS (RAPD-derived) | B5-1520 (SCAR), B5Rlm6_1 (SCAR), BjHZ-1 (CAPS), BnHZ_2 (CAPS) | Primers only | Rashid et al. 2018 |
| *LMJR1*^1^ | AC Vulcan | Resistant lines crossed with inbred line UM3132 (susceptible), segregation population (F2) | RFLP, SSR (microsatellite) | PN199RV (RFLP), sB31143F (SSR) | PN199RV only | Christianson et al. 2006 |
| *LMJR2*^1^ | AC Vulcan | Resistant lines crossed with inbred line UM3132 (susceptible), segregation population (F2) | RFLP, SSR | PN120cRI (RFLP), sB1534 (SSR) | PN120cRI only | Christianson et al. 2006 |
| *r_j_lm2* | *B. napus* introgression lines (with genetic material from *B. juncea* cv. Stoke) 980200, 980204 | Resistant *B. juncea* Stoke crossed with *B. oler*acea Vitamina, *B. juncea*-*B. oleracea* line backcrossed with *B. napus* Andor (susceptible); segregation population (F2, selfed) | SCAR (RAPD-derived), SCAR (derived from RGA-derived) | B5-1520 (RAPD-derived SCAR), C5-1000 (RAPD-derived), RGALm (RGA-derived SCAR) | Marker only | Saal et al. 2004, Saal and Struss 2005 |
| *PhR2*^2^ | *B. napus* introgression lines (with genetic material from *B. juncea* cv. Stoke) | Resistant *B. juncea* Stoke crossed with *B. oleracea* Vitamina, *B. juncea*-*B. oleracea* line backcrossed with *B. napus* Andor (susceptible); segregating population (F2) | RFLPs, STS (RFLP-derived), AFLPs, STS (AFLP-derived) | S7G4 (AFLP-derived STS), pRP1513 (RFLP-derived STS) | Marker sequences for resistant and susceptible | Plieske and Struss 2001 |
|  | *B. napus* introgression lines (with genetic material from *B. juncea* cv. Stoke) | Resistant *B. juncea* Stoke crossed with *B. oleracea* Vitamina, *B. juncea*-*B. oleracea* line backcrossed with *B. napus* Andor (susceptible); segregating population (F2) | RAPD, RFLPs | pRP1457.H, pRP1513.E, pRP1602.H (RFLP) | No | Plieske et al. 1998 |
| Un-named locus | *B. napus* introgression lines (with genetic material from *B. juncea* cv. Stoke) | Resistant *B. juncea* Stoke crossed with *B. oleracea* Vitamina*, B. juncea-B. oleracea* line backcrossed with *B. napus* Andor (susceptible); monosomic addition lines | Isozymes, RAPDs, RFLPs | OPU9 (RAPD) | Decamer primer only | Struss et al. 1996 |
| Three resistance loci | *B. juncea* rapid cycling line | Recombinant lines (*B. napus –B. juncea*) | RFLP | N.A. | No | Dixelius and Wahlberg 1999 |
| *LmBR1* | *B. napus* introgression lines (with genetic material from *B. juncea* cv. CrGC no. 4) | Resistant *B. juncea* cv. CrGC no. 4 crossed with *B. napus* Hanna (susceptible); segregating populations (F1, F2, BC7) | RFLP | N.A. (one unnamed RFLP associated to resistance) | No | Dixelius 1999 |

1. SSR (microsatellite) linked for *LMJR1* and *LMJR2* disease loci for blackleg resistance were identified in the same *B. juncea* linkage group as the SSR marker (sBb31143F and sB1534, respectively) identified as the flanking SSR marker due to sequence not being available.
2. *PhR2*: Position. Linked to resistance. 4 RFLP and 3 AFLP markers were linked to resistance gene *PhR2* where 1 RFLP and 1 AFLP were converted to PCR-based STS markers linked to the same resistance gene and mapped to the same position in linkage maps per the original marker. Sequence for both original markers are provided in the paper. STS marker (AFLP-derived) was not amplified by *B. juncea* utilised however was not original *B. juncea* parent for crossing populations. Position. Linked to susceptible genotypes. STS marker (RFLP-derived) was amplified by *B. juncea* although not original *B. juncea* parent for populations. RFLP marker was absent in *B. juncea* genome.
