# Supplementary material for: In silico integration of disease resistance QTL, genes and markers with the Brassica juncea physical map
Source: Mol Breed. 2022 Jun 27;42(7):37. doi: 10.1007/s11032-022-01309-5 (PMC10248627; doi:10.1007/s11032-022-01309-5)
Supplement: Supplementary file 6 — Supplementary file6 (PDF 289 KB) [file 11032_2022_1309_MOESM6_ESM.pdf]

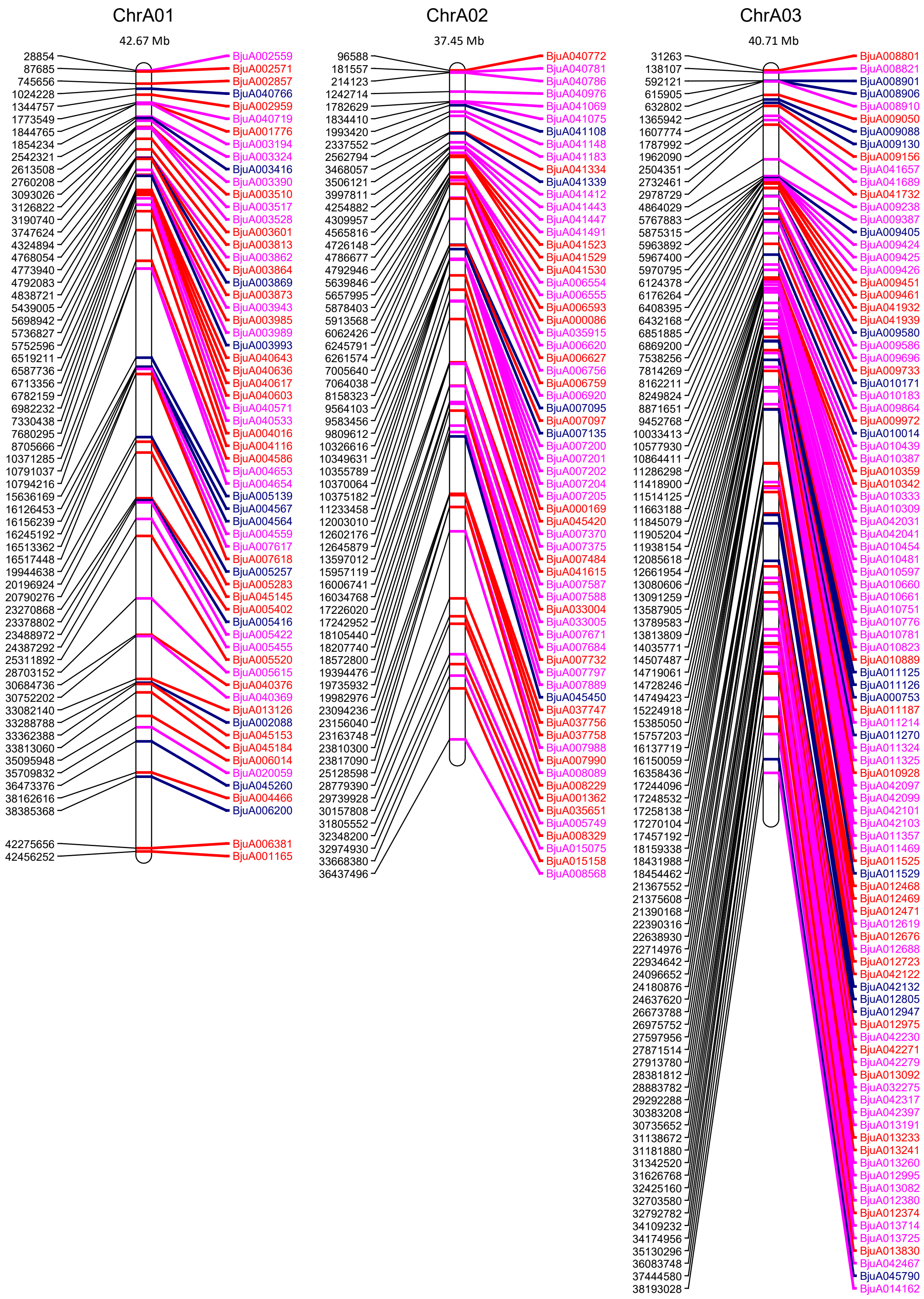
ChrA01
42.67 Mb
28854
87685
745656
1024228
1344757
1773549
1844765
1854234
2542321
2613508
2760208
3093026
3126822
3190740
3747624
4324894
4768054
4773940
4792083
4838721
5439005
5698942
5736827
5752596
6519211
6587736
6713356
6782159
6982232
7330438
7680295
8705666
10371285
10791037
10794216
15636169
16126453
16156239
16245192
16513362
16517448
19944638
20196924
20790276
23270868
23378802
23488972
24387292
25311892
28703152
30684736
30752202
33082140
33288788
33362388
33813060
35095948
35709832
36473376
38162616
38385368
42275656
42456252
BjuA002559
BjuA002571
BjuA002857
BjuA040766
BjuA002959
BjuA040719
BjuA001776
BjuA003194
BjuA003324
BjuA003416
BjuA003390
BjuA003510
BjuA003517
BjuA003528
BjuA003601
BjuA003813
BjuA003862
BjuA003864
BjuA003869
BjuA003873
BjuA003943
BjuA003985
BjuA003989
BjuA003993
BjuA040643
BjuA040636
BjuA040617
BjuA040603
BjuA040571
BjuA040533
BjuA040016
BjuA004116
BjuA004586
BjuA004653
BjuA004654
BjuA005139
BjuA004567
BjuA004564
BjuA004559
BjuA007617
BjuA007618
BjuA005257
BjuA005283
BjuA045145
BjuA005402
BjuA005416
BjuA005422
BjuA005455
BjuA005520
BjuA005615
BjuA040376
BjuA040369
BjuA013126
BjuA002088
BjuA045153
BjuA045184
BjuA006014
BjuA020059
BjuA045260
BjuA004466
BjuA006200
BjuA006381
BjuA001165
ChrA02
37.45 Mb
96588
181557
214123
1242714
1782629
1834410
1993420
2337552
2562794
3468057
3506121
3997811
4254882
4309957
4565816
4726148
4786677
4792946
5639846
5657995
5878403
5913568
6062426
6245791
6261574
7005640
7064038
8158323
9564103
9583456
9809612
10326616
10349631
10355789
10370064
10375182
11233458
12003010
12602176
12645879
13597012
15957119
16006741
16034768
17226020
17242952
18105440
18207740
18572800
19394476
19735932
19982976
23094236
23156040
23163748
23810300
23817090
25128598
28779390
29739928
30157808
31805552
32348200
32974930
33668380
36437496
BjuA040772
BjuA040781
BjuA040786
BjuA040976
BjuA041069
BjuA041075
BjuA041108
BjuA041148
BjuA041183
BjuA041334
BjuA041339
BjuA041412
BjuA041443
BjuA041447
BjuA041491
BjuA041523
BjuA041529
BjuA041530
BjuA006554
BjuA006555
BjuA006593
BjuA000086
BjuA035915
BjuA006620
BjuA006627
BjuA006756
BjuA006759
BjuA006920
BjuA007095
BjuA007097
BjuA007135
BjuA007200
BjuA007201
BjuA007202
BjuA007204
BjuA007205
BjuA000169
BjuA045420
BjuA007370
BjuA007375
BjuA007484
BjuA041615
BjuA007587
BjuA007588
BjuA033004
BjuA033005
BjuA007671
BjuA007684
BjuA007732
BjuA007797
BjuA007889
BjuA045450
BjuA037747
BjuA037756
BjuA037758
BjuA007988
BjuA007990
BjuA008089
BjuA008229
BjuA001362
BjuA035651
BjuA005749
BjuA008329
BjuA015075
BjuA015158
BjuA008568
ChrA03
40.71 Mb
31263
138107
592121
615905
632802
1365942
1607774
1787992
1962090
2504351
2732461
2978729
4864029
5767883
5875315
5963892
5967400
5970795
6124378
6176264
6408395
6432168
6851885
6869200
7538256
7814269
8162211
8249824
8871651
9452768
10033413
10577930
10864411
11286298
11418900
11514125
11663188
11845079
11905204
11938154
12085618
12661954
13080606
13091259
13587905
13789583
13813809
14035771
14507487
14719061
14728246
14749423
15224918
15385050
15757203
16137719
16150059
16358436
17244096
17248532
17258138
17270104
17457192
18159338
18431988
18454462
21367552
21375608
21390168
22390316
22638930
22714976
22934642
24096652
24180876
24637620
26673788
26975752
27597956
27871514
27913780
28381812
28883782
29292288
30383208
30735652
31138672
31181880
31342520
31626768
32425160
32703580
32792782
34109232
34174956
35130296
36083748
37444580
38193028
BjuA008801
BjuA008821
BjuA008901
BjuA008906
BjuA008910
BjuA009050
BjuA009088
BjuA009130
BjuA009156
BjuA041657
BjuA041689
BjuA041732
BjuA009238
BjuA009387
BjuA009405
BjuA009424
BjuA009425
BjuA009426
BjuA009451
BjuA009461
BjuA041932
BjuA041939
BjuA009580
BjuA009586
BjuA009696
BjuA009733
BjuA010171
BjuA010183
BjuA009864
BjuA009972
BjuA010014
BjuA010439
BjuA010387
BjuA010359
BjuA010342
BjuA010333
BjuA010309
BjuA042031
BjuA042041
BjuA010454
BjuA010481
BjuA010597
BjuA010660
BjuA010661
BjuA010751
BjuA010776
BjuA010781
BjuA010823
BjuA010889
BjuA011125
BjuA011126
BjuA000753
BjuA011187
BjuA011214
BjuA011270
BjuA011324
BjuA011325
BjuA010928
BjuA042097
BjuA042099
BjuA042101
BjuA042103
BjuA011357
BjuA011469
BjuA011525
BjuA011529
BjuA012468
BjuA012469
BjuA012471
BjuA012619
BjuA012676
BjuA012688
BjuA012723
BjuA042122
BjuA042132
BjuA012805
BjuA012947
BjuA012975
BjuA042230
BjuA042271
BjuA042279
BjuA013092
BjuA032275
BjuA042317
BjuA042397
BjuA013191
BjuA013233
BjuA013241
BjuA013260
BjuA012995
BjuA013082
BjuA012380
BjuA012374
BjuA013714
BjuA013725
BjuA013830
BjuA042467
BjuA045790
BjuA014162

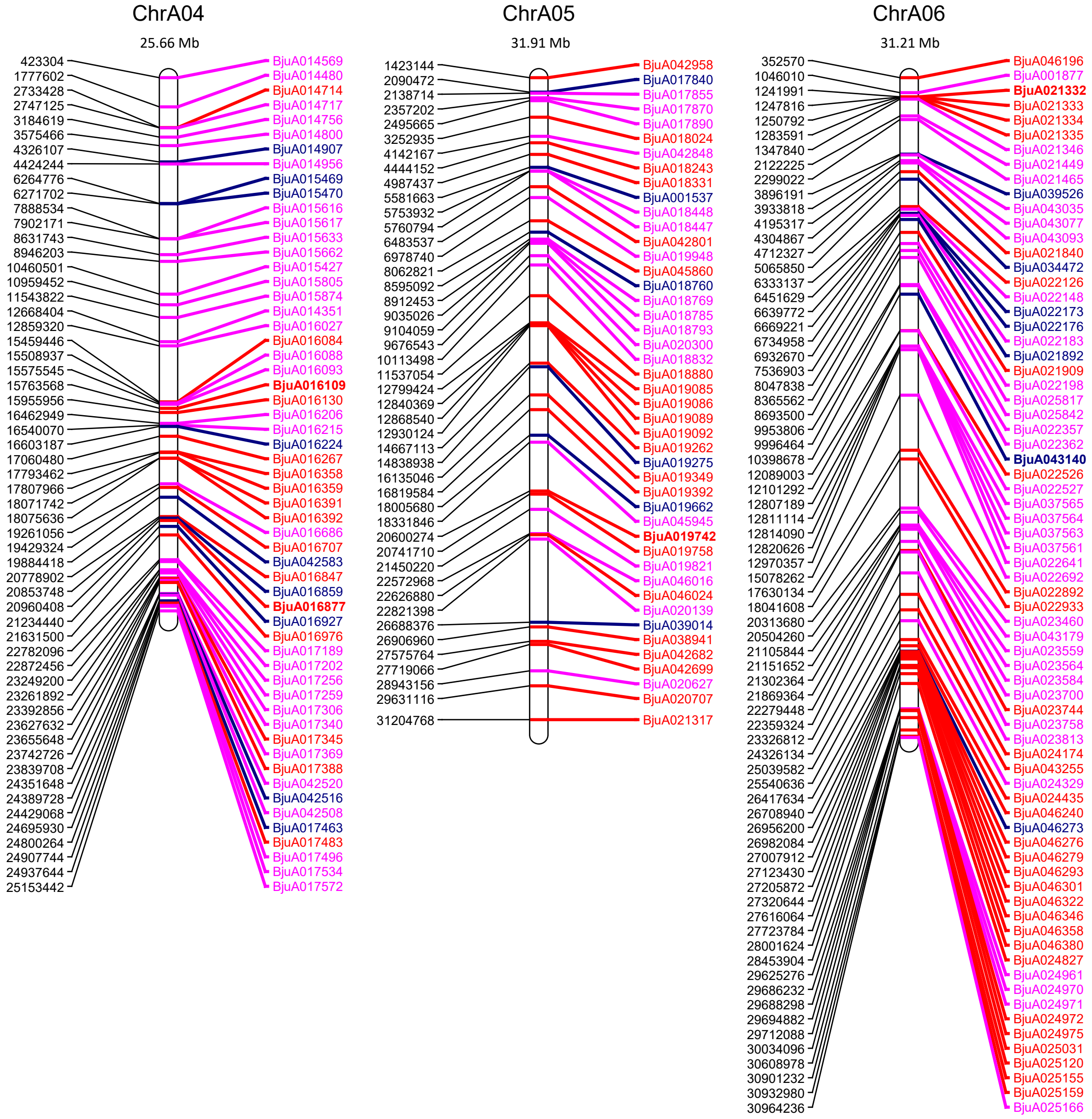
ChrA04
25.66 Mb
423304
1777602
2733428
2747125
3184619
3575466
4326107
4424244
6264776
6271702
7888534
7902171
8631743
8946203
10460501
10959452
11543822
12668404
12859320
15459446
15508937
15575545
15763568
15955956
16462949
16540070
16603187
17060480
17793462
17807966
18071742
18075636
19261056
19429324
19884418
20778902
20853748
20960408
21234440
21631500
22782096
22872456
23249200
23261892
23392856
23627632
23655648
23742726
23839708
24351648
24389728
24429068
24695930
24800264
24907744
24937644
25153442
BjuA014569
BjuA014480
BjuA014714
BjuA014717
BjuA014756
BjuA014800
BjuA014907
BjuA014956
BjuA015469
BjuA015470
BjuA015616
BjuA015617
BjuA015633
BjuA015662
BjuA015427
BjuA015805
BjuA015874
BjuA014351
BjuA016027
BjuA016084
BjuA016088
BjuA016093
BjuA016109
BjuA016130
BjuA016206
BjuA016215
BjuA016224
BjuA016267
BjuA016358
BjuA016359
BjuA016391
BjuA016392
BjuA016686
BjuA016707
BjuA042583
BjuA016847
BjuA016859
BjuA016877
BjuA016927
BjuA016976
BjuA017189
BjuA017202
BjuA017256
BjuA017259
BjuA017306
BjuA017340
BjuA017345
BjuA017369
BjuA017388
BjuA042520
BjuA042516
BjuA042508
BjuA017463
BjuA017483
BjuA017496
BjuA017534
BjuA017572
ChrA05
31.91 Mb
1423144
2090472
2138714
2357202
2495665
3252935
4142167
4444152
4987437
5581663
5753932
5760794
6483537
6978740
8062821
8595092
8912453
9035026
9104059
9676543
10113498
11537054
12799424
12840369
12868540
12930124
14667113
14838938
16135046
16819584
18005680
18331846
20600274
20741710
21450220
22572968
22626880
22821398
26688376
26906960
27575764
27719066
28943156
29631116
31204768
BjuA042958
BjuA017840
BjuA017855
BjuA017870
BjuA017890
BjuA018024
BjuA042848
BjuA018243
BjuA018331
BjuA001537
BjuA018448
BjuA018447
BjuA042801
BjuA019948
BjuA045860
BjuA018760
BjuA018769
BjuA018785
BjuA018793
BjuA020300
BjuA018832
BjuA018880
BjuA019085
BjuA019086
BjuA019089
BjuA019092
BjuA019262
BjuA019275
BjuA019349
BjuA019392
BjuA019662
BjuA045945
BjuA019742
BjuA019758
BjuA019821
BjuA046016
BjuA046024
BjuA020139
BjuA039014
BjuA038941
BjuA042682
BjuA042699
BjuA020627
BjuA020707
BjuA021317
ChrA06
31.21 Mb
352570
1046010
1241991
1247816
1250792
1283591
1347840
2122225
2299022
3896191
3933818
4195317
4304867
4712327
5065850
6333137
6451629
6639772
6669221
6734958
6932670
7536903
8047838
8365562
8693500
9953806
9996464
10398678
12089003
12101292
12807189
12811114
12814090
12820626
12970357
15078262
17630134
18041608
20313680
20504260
21105844
21151652
21302364
21869364
22279448
22359324
23326812
24326134
25039582
25540636
26417634
26708940
26956200
26982084
27007912
27123430
27205872
27320644
27616064
27723784
28001624
28453904
29625276
29686232
29688298
29694882
29712088
30034096
30608978
30901232
30932980
30964236
BjuA046196
BjuA001877
BjuA021332
BjuA021333
BjuA021334
BjuA021335
BjuA021346
BjuA021449
BjuA021465
BjuA039526
BjuA043035
BjuA043077
BjuA043093
BjuA021840
BjuA034472
BjuA022126
BjuA022148
BjuA022173
BjuA022176
BjuA022183
BjuA021892
BjuA021909
BjuA022198
BjuA025817
BjuA025842
BjuA022357
BjuA022362
BjuA043140
BjuA022526
BjuA022527
BjuA037565
BjuA037564
BjuA037563
BjuA037561
BjuA022641
BjuA022692
BjuA022892
BjuA022933
BjuA023460
BjuA043179
BjuA023559
BjuA023564
BjuA023584
BjuA023700
BjuA023744
BjuA023758
BjuA023813
BjuA024174
BjuA043255
BjuA024329
BjuA024435
BjuA046240
BjuA046273
BjuA046276
BjuA046279
BjuA046293
BjuA046301
BjuA046322
BjuA046346
BjuA046358
BjuA046380
BjuA024827
BjuA024961
BjuA024970
BjuA024971
BjuA024972
BjuA024975
BjuA025031
BjuA025120
BjuA025155
BjuA025159
BjuA025166

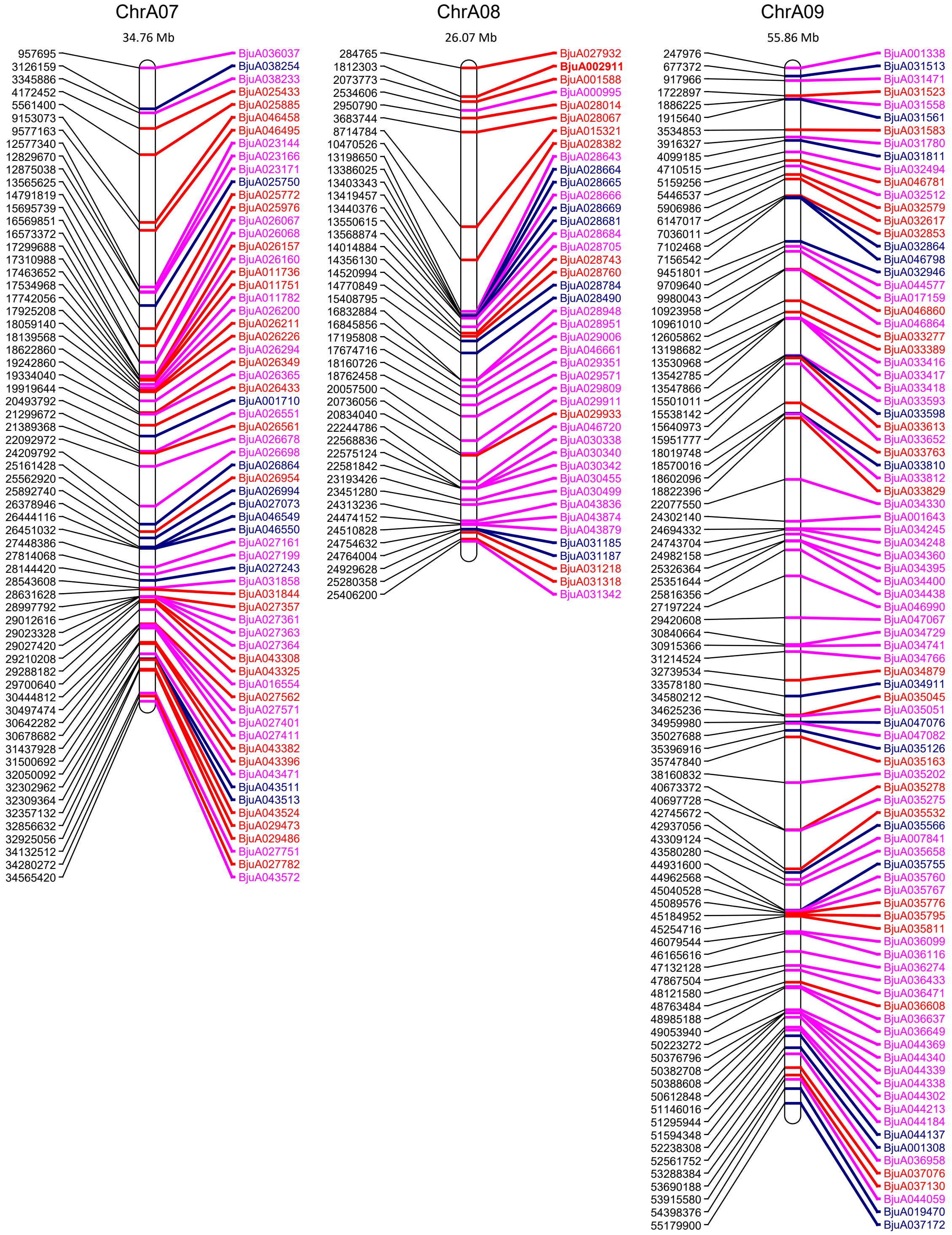
ChrA07
34.76 Mb
957695
3126159
3345886
4172452
5561400
9153073
9577163
12577340
12829670
12875038
13565625
14791819
15695739
16569851
16573372
17299688
17310988
17463652
17534968
17742056
17925208
18059140
18139568
18622860
19242860
19334040
19919644
20493792
21299672
21389368
22092972
24209792
25161428
25562920
25892740
26378946
26444116
26451032
27448386
27814068
28144420
28543608
28631628
28997792
29012616
29023328
29027420
29210208
29288182
29700640
30444812
30497474
30642282
30678682
31437928
31500692
32050092
32302962
32309364
32357132
32856632
32925056
34132512
34280272
34565420
BjuA036037
BjuA038254
BjuA038233
BjuA025433
BjuA025885
BjuA046458
BjuA046495
BjuA023144
BjuA023166
BjuA023171
BjuA025750
BjuA025772
BjuA025976
BjuA026067
BjuA026068
BjuA026157
BjuA026160
BjuA011736
BjuA011751
BjuA011782
BjuA026200
BjuA026211
BjuA026226
BjuA026294
BjuA026349
BjuA026365
BjuA026433
BjuA001710
BjuA026551
BjuA026561
BjuA026678
BjuA026698
BjuA026864
BjuA026954
BjuA026994
BjuA027073
BjuA046549
BjuA046550
BjuA027161
BjuA027199
BjuA027243
BjuA031858
BjuA031844
BjuA027357
BjuA027361
BjuA027363
BjuA027364
BjuA043308
BjuA043325
BjuA016554
BjuA027562
BjuA027571
BjuA027401
BjuA027411
BjuA043382
BjuA043396
BjuA043471
BjuA043511
BjuA043513
BjuA043524
BjuA029473
BjuA029486
BjuA027751
BjuA027782
BjuA043572
ChrA08
26.07 Mb
284765
1812303
2073773
2534606
2950790
3683744
8714784
10470526
13198650
13386025
13403343
13419457
13440376
13550615
13568874
14014884
14356130
14520994
14770849
15408795
16832884
16845856
17195808
17674716
18160726
18762458
20057500
20736056
20834040
22244786
22568836
22575124
22581842
23193426
23451280
24313236
24474152
24510828
24754632
24764004
24929628
25280358
25406200
BjuA027932
BjuA002911
BjuA001588
BjuA000995
BjuA028014
BjuA028067
BjuA015321
BjuA028382
BjuA028643
BjuA028664
BjuA028665
BjuA028666
BjuA028669
BjuA028681
BjuA028684
BjuA028705
BjuA028743
BjuA028760
BjuA028784
BjuA028490
BjuA028948
BjuA028951
BjuA029006
BjuA046661
BjuA029351
BjuA029571
BjuA029809
BjuA029911
BjuA029933
BjuA046720
BjuA030338
BjuA030340
BjuA030342
BjuA030455
BjuA030499
BjuA043836
BjuA043874
BjuA043879
BjuA031185
BjuA031187
BjuA031218
BjuA031318
BjuA031342
ChrA09
55.86 Mb
247976
677372
917966
1722897
1886225
1915640
3534853
3916327
4099185
4710515
5159256
5446537
5906986
6147017
7036011
7102468
7156542
9451801
9709640
9980043
10923958
10961010
12605862
13198682
13530968
13542785
13547866
15501011
15538142
15640973
15951777
18019748
18570016
18602096
18822396
22077550
24302140
24694332
24743704
24982158
25326364
25351644
25816356
27197224
29420608
30840664
30915366
31214524
32739534
33578180
34580212
34625236
34959980
35027688
35396916
35747840
38160832
40673372
40697728
42745672
42937056
43309124
43580280
44931600
44962568
45040528
45089576
45184952
45254716
46079544
46165616
47132128
47867504
48121580
48763484
48985188
49053940
50223272
50376796
50382708
50388608
50612848
51146016
51295944
51594348
52238308
52561752
53288384
53690188
53915580
54398376
55179900
BjuA001338
BjuA031513
BjuA031471
BjuA031523
BjuA031558
BjuA031561
BjuA031583
BjuA031780
BjuA031811
BjuA032494
BjuA046781
BjuA032512
BjuA032579
BjuA032617
BjuA032853
BjuA032864
BjuA046798
BjuA032946
BjuA044577
BjuA017159
BjuA046860
BjuA046864
BjuA033277
BjuA033389
BjuA033416
BjuA033417
BjuA033418
BjuA033593
BjuA033598
BjuA033613
BjuA033652
BjuA033763
BjuA033810
BjuA033812
BjuA033829
BjuA034330
BjuA001643
BjuA034245
BjuA034248
BjuA034360
BjuA034395
BjuA034400
BjuA034438
BjuA046990
BjuA047067
BjuA034729
BjuA034741
BjuA034766
BjuA034879
BjuA034911
BjuA035045
BjuA035051
BjuA047076
BjuA047082
BjuA035126
BjuA035163
BjuA035202
BjuA035278
BjuA035275
BjuA035532
BjuA035566
BjuA007841
BjuA035658
BjuA035755
BjuA035760
BjuA035767
BjuA035776
BjuA035795
BjuA035811
BjuA036099
BjuA036116
BjuA036274
BjuA036433
BjuA036471
BjuA036608
BjuA036637
BjuA036649
BjuA044369
BjuA044340
BjuA044339
BjuA044338
BjuA044302
BjuA044213
BjuA044184
BjuA044137
BjuA001308
BjuA036958
BjuA037076
BjuA037130
BjuA044059
BjuA019470
BjuA037172

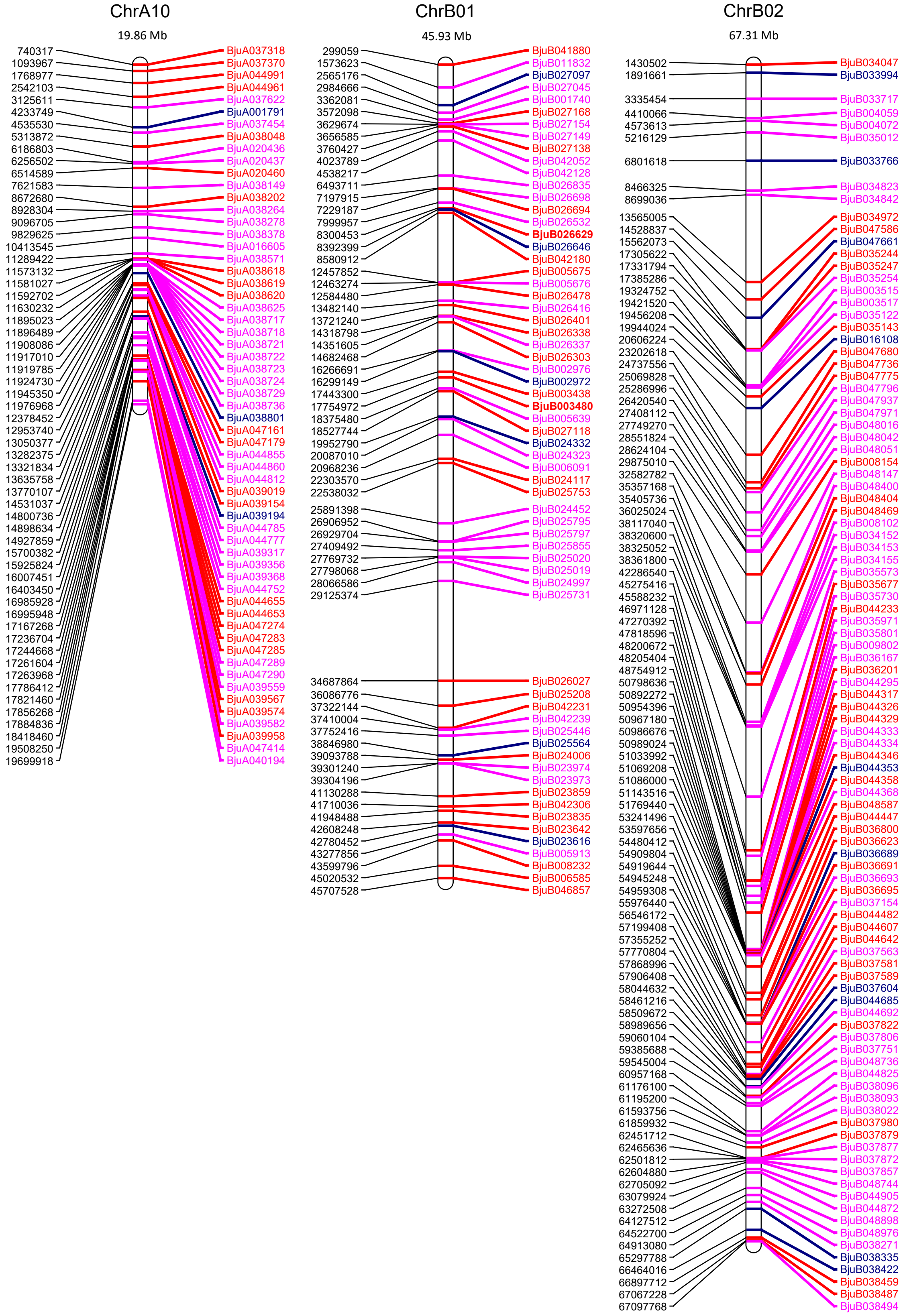

ChrA10
19.86 Mb
740317
1093967
1768977
2542103
3125611
4233749
4535530
5313872
6186803
6256502
6514589
7621583
8672680
8928304
9096705
9829625
10413545
11289422
11573132
11581027
11592702
11630232
11895023
11896489
11908086
11917010
11919785
11924730
11945350
11976968
12378452
12953740
13050377
13282375
13321834
13635758
13770107
14531037
14800736
14898634
14927859
15700382
15925824
16007451
16403450
16985928
16995948
17167268
17236704
17244668
17261604
17263968
17786412
17821460
17856268
17884836
18418460
19508250
19699918
BjuA037318
BjuA037370
BjuA044991
BjuA044961
BjuA037622
BjuA001791
BjuA037454
BjuA038048
BjuA020436
BjuA020437
BjuA020460
BjuA038149
BjuA038202
BjuA038264
BjuA038278
BjuA038378
BjuA016605
BjuA038571
BjuA038618
BjuA038619
BjuA038620
BjuA038625
BjuA038717
BjuA038718
BjuA038721
BjuA038722
BjuA038723
BjuA038724
BjuA038729
BjuA038736
BjuA038801
BjuA047161
BjuA047179
BjuA044855
BjuA044860
BjuA044812
BjuA039019
BjuA039154
BjuA039194
BjuA044785
BjuA044777
BjuA039317
BjuA039356
BjuA039368
BjuA044752
BjuA044655
BjuA044653
BjuA047274
BjuA047283
BjuA047285
BjuA047289
BjuA047290
BjuA039559
BjuA039567
BjuA039574
BjuA039582
BjuA039958
BjuA047414
BjuA040194
ChrB01
45.93 Mb
299059
1573623
2565176
2984666
3362081
3572098
3629674
3656585
3760427
4023789
4538217
6493711
7197915
7229187
7999957
8300453
8392399
8580912
12457852
12463274
12584480
13482140
13721240
14318798
14351605
14682468
16266691
16299149
17443300
17754972
18375480
18527744
19952790
20087010
20968236
22303570
22538032
25891398
26906952
26929704
27409492
27769732
27798068
28066586
29125374
34687864
36086776
37322144
37410004
37752416
38846980
39093788
39301240
39304196
41130288
41710036
41948488
42608248
42780452
43277856
43599796
45020532
45707528
BjuB041880
BjuB011832
BjuB027097
BjuB027045
BjuB001740
BjuB027168
BjuB027154
BjuB027149
BjuB027138
BjuB042052
BjuB042128
BjuB026835
BjuB026698
BjuB026694
BjuB026532
BjuB026629
BjuB026646
BjuB042180
BjuB005675
BjuB005676
BjuB026478
BjuB026416
BjuB026401
BjuB026338
BjuB026337
BjuB026303
BjuB002976
BjuB002972
BjuB003438
BjuB003480
BjuB005639
BjuB027118
BjuB024332
BjuB024323
BjuB006091
BjuB024117
BjuB025753
BjuB024452
BjuB025795
BjuB025797
BjuB025855
BjuB025020
BjuB025019
BjuB024997
BjuB025731
BjuB026027
BjuB025208
BjuB042231
BjuB042239
BjuB025446
BjuB025564
BjuB024006
BjuB023974
BjuB023973
BjuB023859
BjuB042306
BjuB023835
BjuB023642
BjuB023616
BjuB005913
BjuB008232
BjuB006585
BjuB046857
ChrB02
67.31 Mb
1430502
1891661
3335454
4410066
4573613
5216129
6801618
8466325
8699036
13565005
14528837
15562073
17305622
17331794
17385286
19324752
19421520
19456208
19944024
20606224
23202618
24737556
25069828
25286996
26420540
27408112
27749270
28551824
28624104
29875010
32582782
35357168
35405736
36025024
38117040
38320600
38325052
38361800
42286540
45275416
45588232
46971128
47270392
47818596
48200672
48205404
48754912
50798636
50892272
50954396
50967180
50986676
50989024
51033992
51069208
51086000
51143516
51769440
53241496
53597656
54480412
54909804
54919644
54945248
54959308
55976440
56546172
57199408
57355252
57770804
57868996
57906408
58044632
58461216
58509672
58989656
59060104
59385688
59545004
60957168
61176100
61195200
61593756
61859932
62451712
62465636
62501812
62604880
62705092
63079924
63272508
64127512
64522700
64913080
65297788
66464016
66897712
67067228
67097768
BjuB034047
BjuB033994
BjuB033717
BjuB004059
BjuB004072
BjuB035012
BjuB033766
BjuB034823
BjuB034842
BjuB034972
BjuB047586
BjuB047661
BjuB035244
BjuB035247
BjuB035254
BjuB003515
BjuB003517
BjuB035122
BjuB035143
BjuB016108
BjuB047680
BjuB047736
BjuB047775
BjuB047796
BjuB047937
BjuB047971
BjuB048016
BjuB048042
BjuB048051
BjuB008154
BjuB048147
BjuB048400
BjuB048404
BjuB048469
BjuB008102
BjuB034152
BjuB034153
BjuB034155
BjuB035573
BjuB035677
BjuB035730
BjuB044233
BjuB035971
BjuB035801
BjuB009802
BjuB036167
BjuB036201
BjuB044295
BjuB044317
BjuB044326
BjuB044329
BjuB044333
BjuB044334
BjuB044346
BjuB044353
BjuB044358
BjuB044368
BjuB048587
BjuB044447
BjuB036800
BjuB036623
BjuB036689
BjuB036691
BjuB036693
BjuB036695
BjuB037154
BjuB044482
BjuB044607
BjuB044642
BjuB037563
BjuB037581
BjuB037589
BjuB037604
BjuB044685
BjuB044692
BjuB037822
BjuB037806
BjuB037751
BjuB048736
BjuB044825
BjuB038096
BjuB038093
BjuB038022
BjuB037980
BjuB037879
BjuB037877
BjuB037872
BjuB037857
BjuB048744
BjuB044905
BjuB044872
BjuB048898
BjuB048976
BjuB038271
BjuB038335
BjuB038422
BjuB038459
BjuB038487
BjuB038494

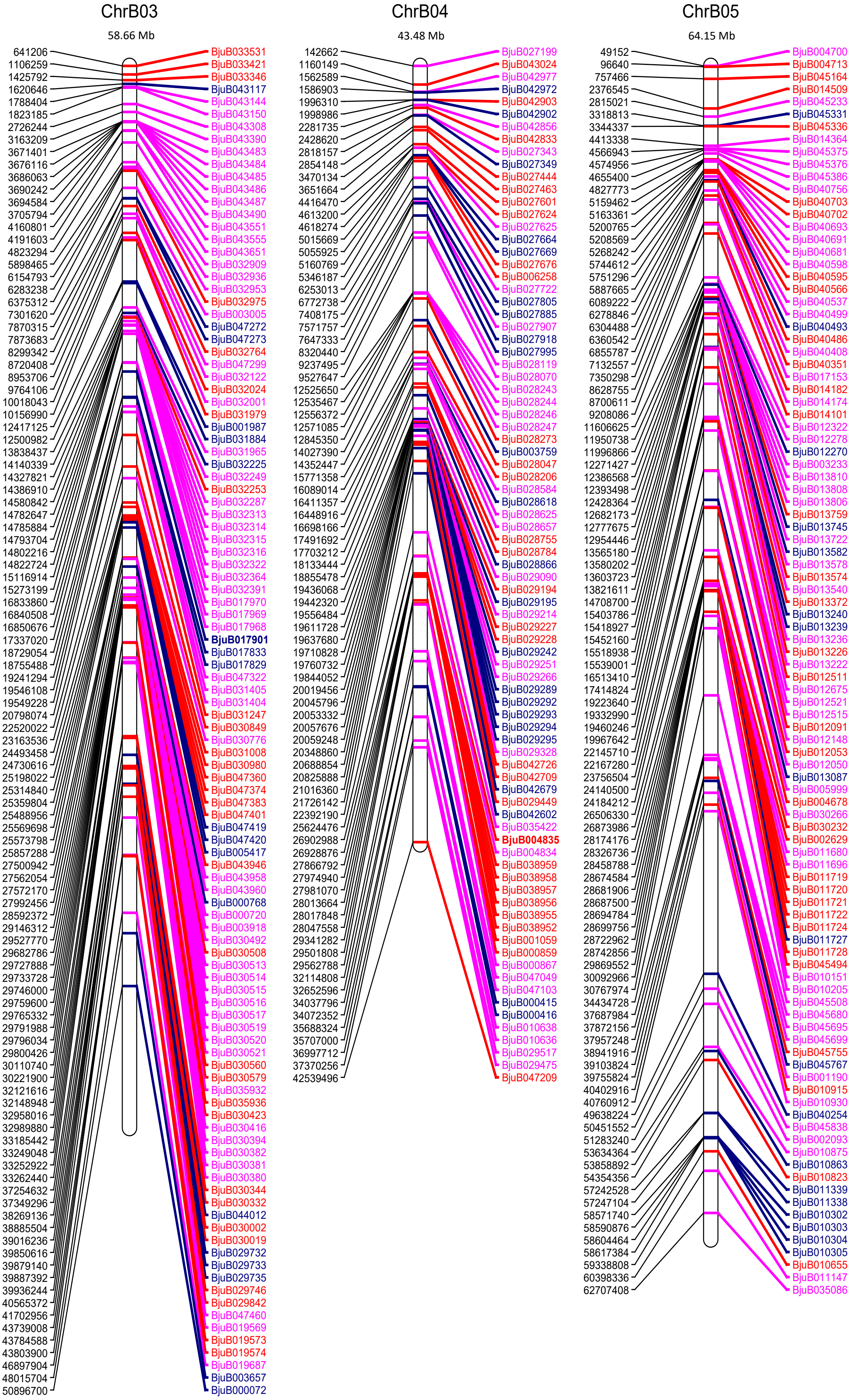
ChrB03
58.66 Mb
641206
1106259
1425792
1620646
1788404
1823185
2726244
3163209
3671401
3676116
3686063
3690242
3694584
3705794
4160801
4191603
4823294
5898465
6154793
6283238
6375312
7301620
7870315
7873683
8299342
8720408
8953706
9764106
10018043
10156990
12417125
12500982
13838437
14140339
14327821
14386910
14580842
14782647
14785884
14793704
14802216
14822724
15116914
15273199
16833860
16840508
16850676
17337020
18729054
18755488
19241294
19546108
19549228
20798074
22520022
23163536
24493458
24730616
25198022
25314840
25359804
25488956
25569698
25573798
25857288
27500942
27562054
27572170
27992456
28592372
29146312
29527770
29682786
29727888
29733728
29746000
29759600
29765332
29791988
29796034
29800426
30110740
30221900
32121616
32148948
32958016
32989880
33185442
33249048
33252922
33262440
37254632
37349296
38269136
38885504
39016236
39850616
39879140
39887392
39936244
40565372
41702956
43739008
43784588
43803900
46897904
48015704
50896700
BjuB033531
BjuB033421
BjuB033346
BjuB043117
BjuB043144
BjuB043150
BjuB043308
BjuB043390
BjuB043483
BjuB043484
BjuB043485
BjuB043486
BjuB043487
BjuB043490
BjuB043551
BjuB043555
BjuB043651
BjuB032909
BjuB032936
BjuB032953
BjuB032975
BjuB003005
BjuB047272
BjuB047273
BjuB032764
BjuB047299
BjuB032122
BjuB032024
BjuB032001
BjuB031979
BjuB001987
BjuB031884
BjuB031965
BjuB032225
BjuB032249
BjuB032253
BjuB032287
BjuB032313
BjuB032314
BjuB032315
BjuB032316
BjuB032322
BjuB032364
BjuB032391
BjuB017970
BjuB017969
BjuB017968
BjuB017901
BjuB017833
BjuB017829
BjuB047322
BjuB031405
BjuB031404
BjuB031247
BjuB030849
BjuB030776
BjuB031008
BjuB030980
BjuB047360
BjuB047374
BjuB047383
BjuB047401
BjuB047419
BjuB047420
BjuB005417
BjuB043946
BjuB043958
BjuB043960
BjuB000768
BjuB000720
BjuB003918
BjuB030492
BjuB030508
BjuB030513
BjuB030514
BjuB030515
BjuB030516
BjuB030517
BjuB030519
BjuB030520
BjuB030521
BjuB030560
BjuB030579
BjuB035932
BjuB035936
BjuB030423
BjuB030416
BjuB030394
BjuB030382
BjuB030381
BjuB030380
BjuB030344
BjuB030332
BjuB044012
BjuB030002
BjuB030019
BjuB029732
BjuB029733
BjuB029735
BjuB029746
BjuB029842
BjuB047460
BjuB019569
BjuB019573
BjuB019574
BjuB019687
BjuB003657
BjuB000072
ChrB04
43.48 Mb
142662
1160149
1562589
1586903
1996310
1998986
2281735
2428620
2818157
2854148
3470134
3651664
4416470
4613200
4618274
5015669
5055925
5160769
5346187
6253013
6772738
7408175
7571757
7647333
8320440
9237495
9527647
12525650
12535467
12556372
12571085
12845350
14027390
14352447
15771358
16089014
16411357
16448916
16698166
17491692
17703212
18133444
18855478
19436068
19442320
19556484
19611728
19637680
19710828
19760732
19844052
20019456
20045796
20053332
20057676
20059248
20348860
20688854
20825888
21016360
21726142
22392190
25624476
26902988
26928876
27866792
27974940
27981070
28013664
28017848
28047558
29341282
29501808
29562788
32114808
32652596
34037796
34072352
35688324
35707000
36997712
37370256
42539496
BjuB027199
BjuB043024
BjuB042977
BjuB042972
BjuB042903
BjuB042902
BjuB042856
BjuB042833
BjuB027343
BjuB027349
BjuB027444
BjuB027463
BjuB027601
BjuB027624
BjuB027625
BjuB027664
BjuB027669
BjuB027676
BjuB006258
BjuB027722
BjuB027805
BjuB027885
BjuB027907
BjuB027918
BjuB027995
BjuB028119
BjuB028070
BjuB028243
BjuB028244
BjuB028246
BjuB028247
BjuB028273
BjuB003759
BjuB028047
BjuB028206
BjuB028584
BjuB028618
BjuB028625
BjuB028657
BjuB028755
BjuB028784
BjuB028866
BjuB029090
BjuB029194
BjuB029195
BjuB029214
BjuB029227
BjuB029228
BjuB029242
BjuB029251
BjuB029266
BjuB029289
BjuB029292
BjuB029293
BjuB029294
BjuB029295
BjuB029328
BjuB042726
BjuB042709
BjuB042679
BjuB029449
BjuB042602
BjuB035422
BjuB004835
BjuB004834
BjuB038959
BjuB038958
BjuB038957
BjuB038956
BjuB038955
BjuB038952
BjuB001059
BjuB000859
BjuB000867
BjuB047049
BjuB047103
BjuB000415
BjuB000416
BjuB010638
BjuB010636
BjuB029517
BjuB029475
BjuB047209
ChrB05
64.15 Mb
49152
96640
757466
2376545
2815021
3318813
3344337
4413338
4566943
4574956
4655400
4827773
5159462
5163361
5200765
5208569
5268242
5744612
5751296
5887665
6089222
6278846
6304488
6360542
6855787
7132557
7350298
8628755
8700611
9208086
11606625
11950738
11996866
12271427
12386568
12393498
12428364
12682173
12777675
12954446
13565180
13580202
13603723
13821611
14708700
15403786
15418927
15452160
15518938
15539001
16513410
17414824
19223640
19332990
19460246
19967642
22145710
22167280
23756504
24140500
24184212
26506330
26873986
28174176
28326736
28458788
28674584
28681906
28687500
28694784
28699756
28722962
28742856
29869552
30092966
30767974
34434728
37687984
37872156
37957248
38941916
39103824
39755824
40402916
40760912
49638224
50451552
51283240
53634364
53858892
54354356
57242528
57247104
58571740
58590876
58604464
58617384
59338808
60398336
62707408
BjuB004700
BjuB004713
BjuB045164
BjuB014509
BjuB045233
BjuB045331
BjuB045336
BjuB014364
BjuB045375
BjuB045376
BjuB045386
BjuB040756
BjuB040703
BjuB040702
BjuB040693
BjuB040691
BjuB040681
BjuB040598
BjuB040595
BjuB040566
BjuB040537
BjuB040499
BjuB040493
BjuB040486
BjuB040408
BjuB040351
BjuB017153
BjuB014182
BjuB014174
BjuB014101
BjuB012322
BjuB012278
BjuB012270
BjuB003233
BjuB013810
BjuB013808
BjuB013806
BjuB013759
BjuB013745
BjuB013722
BjuB013582
BjuB013578
BjuB013574
BjuB013540
BjuB013372
BjuB013240
BjuB013239
BjuB013236
BjuB013226
BjuB013222
BjuB012511
BjuB012675
BjuB012521
BjuB012515
BjuB012091
BjuB012148
BjuB012053
BjuB012050
BjuB013087
BjuB005999
BjuB004678
BjuB030266
BjuB030232
BjuB002629
BjuB011680
BjuB011696
BjuB011719
BjuB011720
BjuB011721
BjuB011722
BjuB011724
BjuB011727
BjuB011728
BjuB045494
BjuB010151
BjuB010205
BjuB045508
BjuB045680
BjuB045695
BjuB045699
BjuB045755
BjuB045767
BjuB001190
BjuB010915
BjuB010930
BjuB040254
BjuB045838
BjuB002093
BjuB010875
BjuB010863
BjuB010823
BjuB011339
BjuB011338
BjuB010302
BjuB010303
BjuB010304
BjuB010305
BjuB010655
BjuB011147
BjuB035086

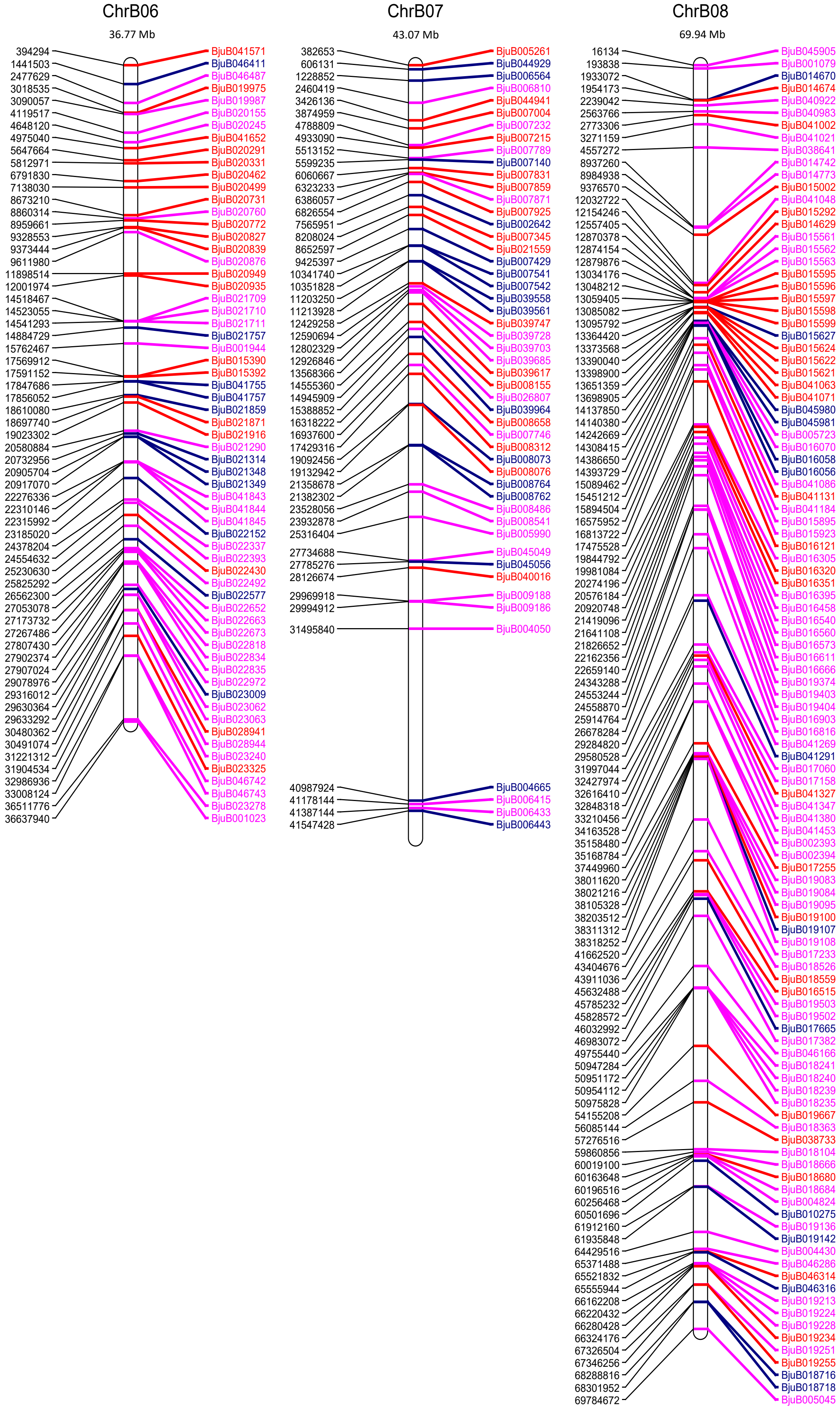
ChrB06
36.77 Mb
394294
1441503
2477629
3018535
3090057
4119517
4648120
4975040
5647664
5812971
6791830
7138030
8673210
8860314
8959661
9328553
9373444
9611980
11898514
12001974
14518467
14523055
14541293
14884729
15762467
17569912
17591152
17847686
17856052
18610080
18697740
19023302
20580884
20732956
20905704
20917070
22276336
22310146
22315992
23185020
24378204
24554632
25230630
25825292
26562300
27053078
27173732
27267486
27807430
27902374
27907024
29078976
29316012
29630364
29633292
30480362
30491074
31221312
31904534
32986936
33008124
36511776
36637940
BjuB041571
BjuB046411
BjuB046487
BjuB019975
BjuB019987
BjuB020155
BjuB020245
BjuB041652
BjuB020291
BjuB020331
BjuB020462
BjuB020499
BjuB020731
BjuB020760
BjuB020772
BjuB020827
BjuB020839
BjuB020876
BjuB020949
BjuB020935
BjuB021709
BjuB021710
BjuB021711
BjuB021757
BjuB001944
BjuB015390
BjuB015392
BjuB041755
BjuB041757
BjuB021859
BjuB021871
BjuB021916
BjuB021290
BjuB021314
BjuB021348
BjuB021349
BjuB041843
BjuB041844
BjuB041845
BjuB022152
BjuB022337
BjuB022393
BjuB022430
BjuB022492
BjuB022577
BjuB022652
BjuB022663
BjuB022673
BjuB022818
BjuB022834
BjuB022835
BjuB022972
BjuB023009
BjuB023062
BjuB023063
BjuB028941
BjuB028944
BjuB023240
BjuB023325
BjuB046742
BjuB046743
BjuB023278
BjuB001023
ChrB07
43.07 Mb
382653
606131
1228852
2460419
3426136
3874959
4788809
4933090
5513152
5599235
6060667
6323233
6386057
6826554
7565951
8208024
8652597
9425397
10341740
10351828
11203250
11213928
12429258
12590694
12802329
12926846
13568366
14555360
14945909
15388852
16318222
16937600
17429316
19092456
19132942
21358678
21382302
23528056
23932878
25316404
27734688
27785276
28126674
29969918
29994912
31495840
40987924
41178144
41387144
41547428
BjuB005261
BjuB044929
BjuB006564
BjuB006810
BjuB044941
BjuB007004
BjuB007232
BjuB007215
BjuB007789
BjuB007140
BjuB007831
BjuB007859
BjuB007871
BjuB007925
BjuB002642
BjuB007345
BjuB021559
BjuB007429
BjuB007541
BjuB007542
BjuB039558
BjuB039561
BjuB039747
BjuB039728
BjuB039703
BjuB039685
BjuB039617
BjuB008155
BjuB026807
BjuB039964
BjuB008658
BjuB007746
BjuB008312
BjuB008073
BjuB008076
BjuB008764
BjuB008762
BjuB008486
BjuB008541
BjuB005990
BjuB045049
BjuB045056
BjuB040016
BjuB009188
BjuB009186
BjuB004050
BjuB004665
BjuB006415
BjuB006433
BjuB006443
ChrB08
69.94 Mb
16134
193838
1933072
1954173
2239042
2563766
2773306
3271159
4557272
8937260
8984938
9376570
12032722
12154246
12557405
12870378
12874154
12879876
13034176
13048212
13059405
13085082
13095792
13364420
13373568
13390040
13398900
13651359
13698905
14137850
14140380
14242669
14308415
14386650
14393729
15089462
15451212
15894504
16575952
16813722
17475528
19844792
19981084
20274196
20576184
20920748
21419096
21641108
21826652
22162356
22659140
24343288
24553244
24558870
25914764
26678284
29284820
29580528
31997044
32427974
32616410
32848318
33210456
34163528
35158480
35168784
37449960
38011620
38021216
38105328
38203512
38311312
38318252
41662520
43404676
43911036
45632488
45785232
45828572
46032992
46983072
49755440
50947284
50951172
50954112
50975828
54155208
56085144
57276516
59860856
60019100
60163648
60196516
60256468
60501696
61912160
61935848
64429516
65371488
65521832
65555944
66162208
66220432
66280428
66324176
67326504
67346256
68288816
68301952
69784672
BjuB045905
BjuB001079
BjuB014670
BjuB014674
BjuB040922
BjuB040983
BjuB041002
BjuB041021
BjuB038641
BjuB014742
BjuB014773
BjuB015002
BjuB041048
BjuB015292
BjuB014629
BjuB015561
BjuB015562
BjuB015563
BjuB015595
BjuB015596
BjuB015597
BjuB015598
BjuB015599
BjuB015627
BjuB015624
BjuB015622
BjuB015621
BjuB041063
BjuB041071
BjuB045980
BjuB045981
BjuB005723
BjuB016070
BjuB016058
BjuB016056
BjuB041086
BjuB041131
BjuB041184
BjuB015895
BjuB015923
BjuB016121
BjuB016305
BjuB016320
BjuB016351
BjuB016395
BjuB016458
BjuB016540
BjuB016560
BjuB016573
BjuB016611
BjuB016666
BjuB019374
BjuB019403
BjuB019404
BjuB016903
BjuB016816
BjuB041269
BjuB041291
BjuB017060
BjuB017158
BjuB041327
BjuB041347
BjuB041380
BjuB041453
BjuB002393
BjuB002394
BjuB017255
BjuB019083
BjuB019084
BjuB019095
BjuB019100
BjuB019107
BjuB019108
BjuB017233
BjuB018526
BjuB018559
BjuB016515
BjuB019503
BjuB019502
BjuB017665
BjuB017382
BjuB046166
BjuB018241
BjuB018240
BjuB018239
BjuB018235
BjuB019667
BjuB018363
BjuB038733
BjuB018104
BjuB018666
BjuB018680
BjuB018684
BjuB004824
BjuB010275
BjuB019136
BjuB019142
BjuB004430
BjuB046286
BjuB046314
BjuB046316
BjuB019213
BjuB019224
BjuB019228
BjuB019234
BjuB019251
BjuB019255
BjuB018716
BjuB018718
BjuB005045

Supplementary figure 1. Physical mapping and distribution of resistance gene analogs (RGA) on *B. juncea* chromosomes. ChrA01 – ChrB08 represents chromosomes of *B. juncea* in A and B sub-genomes ChrA01 – ChrB08 represented as white bars. The chromosome size is shown above the chromosome bar in Mb. Gene name is shown on the right-hand side and their physical positions in base pair (bp) is shown on the left. Different colours and font of gene name reflect different class of RGA; RLP (Red), RLK (Pink) and NBS (Dark blue) with position in chromosome (bp).
